# SETD2 deficiency accelerates sphingomyelin accumulation and promotes the development of renal cancer

**DOI:** 10.1038/s41467-023-43378-w

**Published:** 2023-11-21

**Authors:** Hanyu Rao, Changwei Liu, Aiting Wang, Chunxiao Ma, Yue Xu, Tianbao Ye, Wenqiong Su, Peijun Zhou, Wei-Qiang Gao, Li Li, Xianting Ding

**Affiliations:** 1 Department of Anesthesiology and Surgical Intensive Care Unit, Xinhua Hospital, School of Medicine and School of Biomedical Engineering, Shanghai Jiao Tong University, Shanghai, China; 2 State Key Laboratory of Systems Medicine for Cancer, Institute for Personalized Medicine and Med-X Research Institute, Shanghai Jiao Tong University, Shanghai, China; 3 State Key Laboratory of Systems Medicine for Cancer, Renji-Med X Clinical Stem Cell Research Center, Ren Ji Hospital, School of Medicine and School of Biomedical Engineering, Shanghai Jiao Tong University, Shanghai, 200127 China; 4 Shanghai Sixth People’s Hospital Affiliated to Shanghai Jiao Tong University School of Medicine, Shanghai, China; 5 Division of Kidney Transplant, Department of Urology, Ruijin Hospital, School of Medicine, Shanghai Jiao Tong University, Shanghai, China

**Keywords:** Renal cell carcinoma, Cancer metabolism, Tumour-suppressor proteins, Proteomic analysis

## Abstract

Patients with polycystic kidney disease (PKD) encounter a high risk of clear cell renal cell carcinoma (ccRCC), a malignant tumor with dysregulated lipid metabolism. SET domain–containing 2 (SETD2) has been identified as an important tumor suppressor and an immunosuppressor in ccRCC. However, the role of SETD2 in ccRCC generation in PKD remains largely unexplored. Herein, we perform metabolomics, lipidomics, transcriptomics and proteomics within SETD2 loss induced PKD-ccRCC transition mouse model. Our analyses show that SETD2 loss causes extensive metabolic reprogramming events that eventually results in enhanced sphingomyelin biosynthesis and tumorigenesis. Clinical ccRCC patient specimens further confirm the abnormal metabolic reprogramming and sphingomyelin accumulation. Tumor symptom caused by *Setd2* knockout is relieved by myriocin, a selective inhibitor of serine-palmitoyl-transferase and sphingomyelin biosynthesis. Our results reveal that SETD2 deficiency promotes large-scale metabolic reprogramming and sphingomyelin biosynthesis during PKD-ccRCC transition. This study introduces high-quality multi-omics resources and uncovers a regulatory mechanism of SETD2 on lipid metabolism during tumorigenesis.

## Abstract (web summary)

SET domain–containing 2 (SETD2) is reported as an immunosuppressor in clear cell renal cell carcinoma (ccRCC). Here the authors show that SETD2 loss enhances de novo sphingomyelin biosynthesis during the transition from polycystic kidney disease to ccRCC.

## Introduction

Polycystic kidney disease (PKD) is the most common kidney disease that is defined by the fluid-filled cysts from renal tubules^1^. PKD patients suffer from a high risk of developing end-stage renal disease, especially renal cell carcinoma (RCC)^2^. RCC is the second leading cause of death among all types of urologic cancers, accounting for >90% of cancers in the kidney^3,4^. Inherited predisposition to RCC has been shown to arise from genes involved in regulating cellular metabolism, indicating a critical role of oncologic-metabolic shift in RCC formation^5^. The most common subtype of RCC is clear cell renal cell carcinoma (ccRCC), which accounts for 75–80% of all diagnosed cases^6^. Morphologically, ccRCC cells are lipid- and glycogen-laden^7^, implicating altered lipid and glucose metabolism in tumor cells, making ccRCC the poster child of malignancies characterized by metabolic reprogramming^8^. The landmark TCGA analysis of ccRCC highlighted a key role of metabolic alteration in ccRCC progression^4^. Oncogenic metabolism has thus emerged as central features of ccRCC.

Histone methyltransferase SET-domain-containing 2 (SETD2) is one of the most frequently mutated genes in ccRCC and plays major roles in epigenetic regulation of functional pathways in the development and progression of ccRCC^9–11^. SETD2 participates in diverse chromatin biological processes, including transcriptional regulation, DNA damage repair, crosstalk of histone modification, alternating splicing and non-histone targets methylation^12^. Mutations or functional loss of SETD2 leads to dysfunctional tumor proteins and further tumorigenesis, progression, chemotherapy resistance and unfavorable prognosis^13^. As a methyltransferase, SETD2 has a capacity to regulate cellular signaling and metabolic responses through epigenetic regulation or modification of proteins^14–16^. However, the molecular mechanism of SETD2 in the transition from PKD to ccRCC remains elusive.

Multi-omics analysis allows for a comprehensive understanding of the molecular mechanisms underlying cancers, and facilitates the discovery of potential therapeutic targets^17^. Herein, we perform systematic multi-omics characterizations on PKD mice (driven by *c-MYC*) and ccRCC mice (driven by *c-MYC* and *Setd2* knockout) to investigate SETD2 function in tumor metabolism during PDK-ccRCC transition. Specifically, we first collect metabolomics and lipidomics data and find sphingomyelin (SM) is the most increased lipid in SETD2 deficient ccRCC. Furthermore, we adopt transcriptomics and proteomics analyses to cross-verify the metabolic alteration after SETD2 deletion on mRNA and protein levels. Our results show that SETD2 loss promotes metabolic reprogramming and eventually leads to enhanced de novo biosynthesis of SM and ccRCC formation. Further spatial metabolomics with clinical ccRCC patient specimens confirm abnormal in situ SM accumulation in humans. This crucial role of SETD2 in lipid metabolism is further validified with in vivo intervention that tumor symptom caused by *Setd2* knockout is mitigated by myriocin, a selective inhibitor for sphingomyelin biosynthesis. Therefore, our findings shed light on roles of the SETD2 in metabolic reprogramming and lipid biosynthesis during the transition from PDK to ccRCC.

## Results

### Multi-omics analysis on SETD2-loss-induced lipid accumulation and PKD-ccRCC transition

**Fig. 1:**
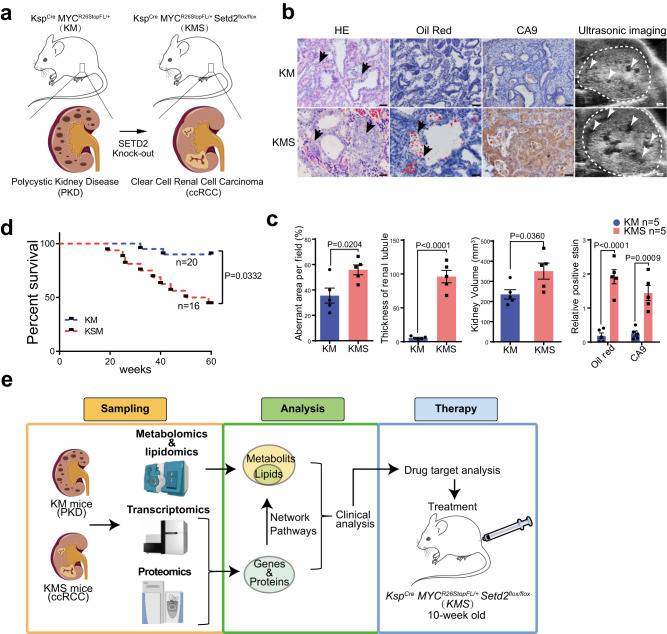
SETD2 loss leads to lipid accumulation and ccRCC generation in PKD mice. **a** Schematic representation of the establishment of PKD and ccRCC mouse model. **b** Representative images of H&E, Oil-red staining and longitudinal ultrasonic testing. Aberrant areas were indicated by arrows. Kidneys were indicated by white dotted lines in ultrasonic images. **c** KMS mice exhibited larger aberrant areas (*P* = 0.0204), thicker renal tubules (*P* < 0.0001), larger kidney volumes (*P* = 0.0360), and more positive staining for Oil red (*P* < 0.0001) and CA9 (*P* = 0.0009). **d** Kaplan–Meier survival curve of indicated mice. **e** General workflow of multi-omics investigations and data analysis. Scale bars, 50 μm. Statistical comparisons were made using a two-tailed Student *t* test. Data are represented as mean ± SEM. Source data are provided as a Source Data file.

To explore potential role of SETD2 in PKD-ccRCC transition in vivo, we generated a PKD mouse model by overexpressing oncogene *c-MYC* under the control of the *Ksp* promoter (hereafter referred as KM mice), and knockout *Setd2* gene using the same *Ksp* promoter in this PKD mice (hereafter referred as KMS mice) to establish a ccRCC mouse model^18^ (Fig. 1a). As shown in Fig. 1b, cysts were observed in both KM and KMS mice at the age of 20 weeks. Notably, KMS mice exhibited obvious structural abnormalities characterized by positive stains of Oil-red-O, a lysochrome diazo dye used for the staining of lipids^19^, and Carboxy anhydrase 9 (CA9), a major marker to diagnose ccRCC^20^, whereas KM mice did not show any neoplastic masses or lipids accumulation (Fig. 1b, c). Meanwhile, the life spans of KMS mice were much shorter compared with KM mice (Fig. 1d). These results demonstrate that SETD2 deletion increases the lipid accumulation and the incidence of ccRCC in PKD mice, highlighting the metabolic regulatory function of SETD2 in the transition from PDK to ccRCC.

To develop a comprehensive molecular understanding of SETD2-loss-induced lipid accumulation and ccRCC generation, we conducted an integrative analysis on SETD2 wild-type PKD mouse model and SETD2 deficient ccRCC mouse model by incorporating multi-omics techniques (Fig. 1e). Specifically, we analyzed metabolomic and lipidomic data first to identify the accumulated lipids. Then, transcriptomic and proteomic data were utilized for regulatory network analysis on metabolic pathways. Human ccRcc specimens were further examined by spatial metabolomics to confirm the abnormal in situ sphingomyelin accumulation. At last, based on multi-omics data, targeted drug was selected for the therapeutic analysis on SETD2 deficient ccRCC mouse model.

### Metabolomic and lipidomic analyses reveal sphingomyelin accumulation in SETD2 deficient ccRCC

**Fig. 2:**
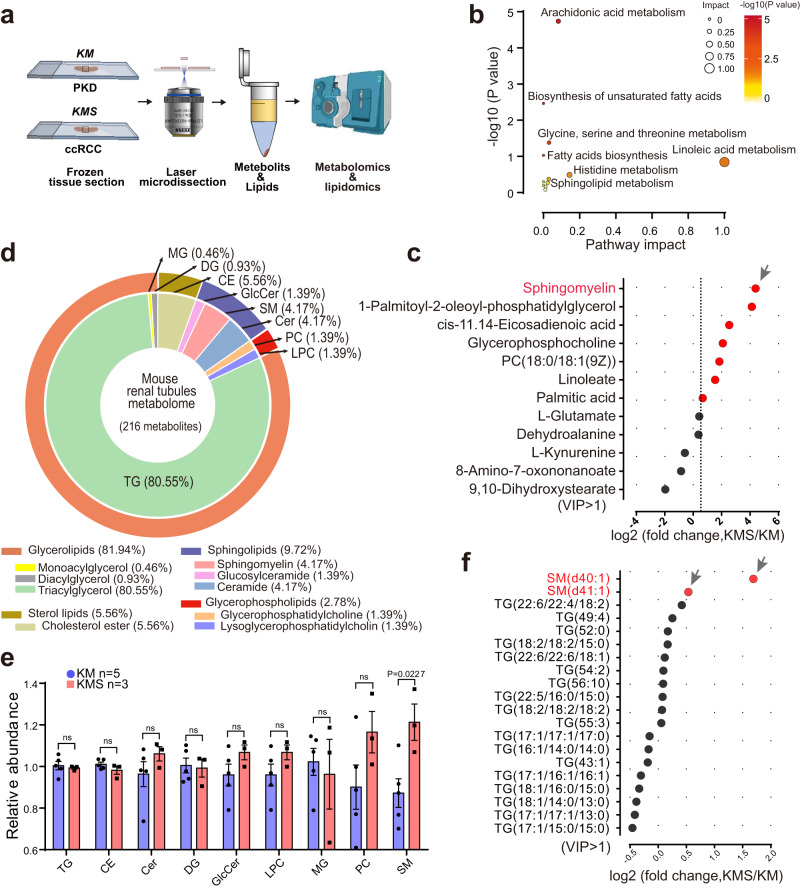
Metabolomics analysis reveals sphingomyelin accumulation in SETD2 deficient ccRCC. **a** Schematic representation of the metabolomics analyses of KM and KMS mice. **b** Altered KEGG metabolic pathways in KMS mice compared to KM mice enriched by significantly altered metabolites (VIP > 1). **c** Differential metabolites between KMS and KM mice (VIP > 1). Top 7 of the most upregulated metabolites are colored in red. **d** Chemical composition of the mouse renal tubules lipidome using ClassyFire categories to classify the lipids diversity of all annotated lipids across assays. **e** Relative abundance of representative lipids between KM (*n* = 5) and KMS (*n* = 3). Statistical comparisons were made using a two-tailed Student *t* test. Data are represented as mean ± SEM. **f** Differential lipids between KMS and KM mice (VIP > 1). Top 2 of the most upregulated lipids are colored in red. Source data are provided as a Source Data file.

To identify the metabolic processes and metabolites influenced by SETD2 deletion during PKD-ccRCC transition, the kidney cysts from KM mice and neoplastic masses from KMS mice were isolated and enriched by laser capture microdissection (LCM). Then liquid chromatography-mass spectrometry (LC-MS)-based untargeted metabolomics and lipidomics analysis were performed on these samples (Fig. 2a). Principal-component analysis (PCA) discriminated SETD2 deficient neoplastic masses from KM controls based on abundance of detected metabolites (Supplementary Fig. 1a). Thirty-five differential metabolites were identified (VIP > 1) (Supplementary Fig. 1b and Supplementary Data 1) and subjected to Kyoto Encyclopedia of Genes and Genomes (KEGG) pathway enrichment analysis. Pathway enrichment data revealed altered fatty acids biosynthesis and amino acid metabolism in SETD2 deficient KMS samples (Fig. 2b). Further, we examined the differential metabolites (VIP > 1) and found that sphingomyelin (SM) was the most prominently upregulated metabolite in KMS samples compared to KM samples (Fig. 2c). SM, a phosphosphingolipid accounting for 5–10% of total phospholipids, is characterized by a sphingoid backbone that is linked with a fatty acid, an amide bond and a phosphorylcholine head group^21^. Remarkably, SM is an essential component of mammalian cells, and regulates signal transduction, such as proliferation/survival, migration, and inflammation, in tumorigenesis^22^.

To identify the specifically increased lipids caused by SETD2 deletion during PKD-ccRCC transition, we next tested untargeted lipid metabolomics data on KM and KMS mouse models. Using the ClassyFire classification system^23^, all lipids were divided into nine lipid subclasses, including Monoacylglycerol (MG, 0.46%), Diacylglycerol (DG, 0.93%), Triacylglycerol (TG, 80.55%), Sphingomyelin (SM, 4.17%), Ceramide (Cer, 4.17%), Glucosylceramide (GlcCer, 1.39%), Cholesterol ester (CE, 5.56%), Glycerophosphatidylcholine (PC, 1.39%) and Lysoglycerophosphatidylcholin (LPC, 1.39%) (Fig. 2d). Only SM was significantly upregulated among these classes of lipids (Fig. 2e). To gain an in-depth insight into the substantially increased lipids, we identified sixty-three differential lipids (VIP > 1) between KMS and KM samples (Supplementary Fig. 1b–d and Supplementary Data 2). In line with our untargeted metabolomics, SM (d40:1) and SM (d41:1) were the most prominently increased lipids in KMS samples compared to KM samples (Fig. 2f). Elevated sphingomyelin levels were also observed upon *Setd2* knockout and the application of the SETD2 inhibitor, SETD2-IN-1^24^, in mouse renal primary tubular epithelial cells (Supplementary Fig. 1e). These results indicate that SETD2 deletion leads to increased content of SM during PKD-ccRCC transition.

### Transcriptomic and proteomic analyses further indicate enhanced metabolic pathways in SETD2 deficient ccRCC

**Fig. 3:**
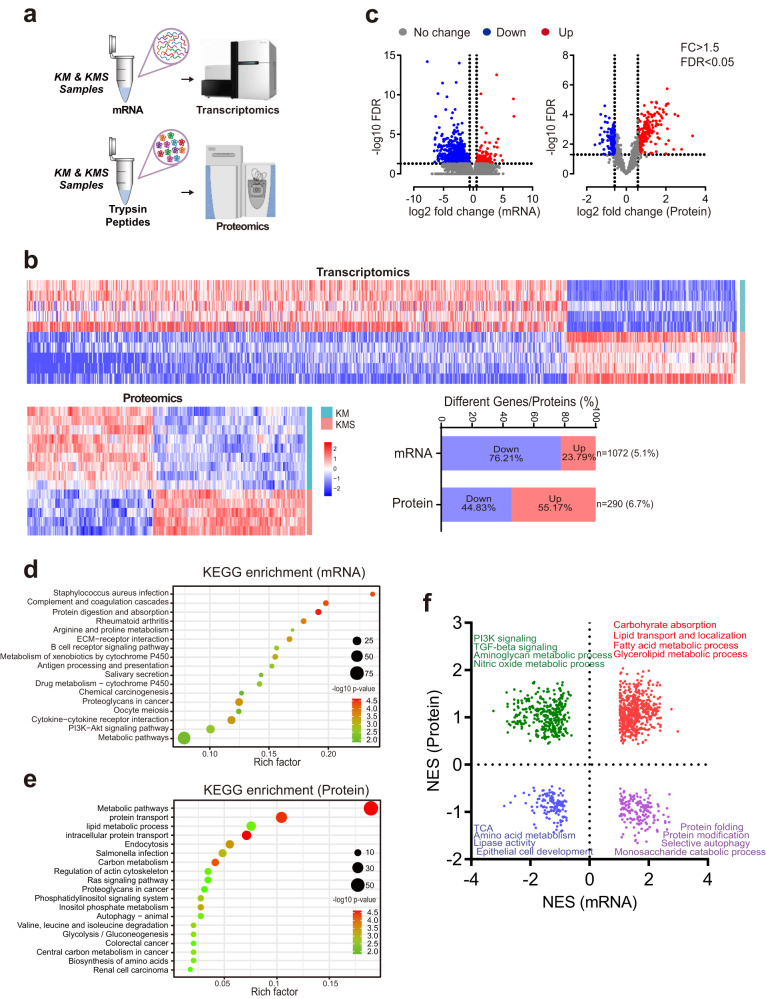
SETD2 deficient ccRCC displays altered metabolic pathways. **a** Schematic representation of the transcriptomics and proteomics analyses of KM and KMS mice. **b** Heat map of the top 290 altered proteins between KM (*n* = 9) and KMS (*n* = 5) mice. Heat map of the top 1072 altered genes between KM (*n* = 5) and KMS (*n* = 5) mice. **c** Volcano plot of gene and protein alterations between KM and KMS mice. Significantly differential proteins (FDR < 0.05 and fold change >1.5) are colored in red (upregulated) and blue (downregulated) in KMS mice compared to KM mice, while others are colored in gray. **d** Significantly altere**d** KEGG pathways between KM and KMS mice based on differential genes. **e** Significantly altered KEGG pathways between KM and KMS mice based on differential proteins. **f** GSEA analysis of upregulated and downregulated pathways in KMS mice compared to KM mice at mRNA (x axis) and protein (y axis) levels based on GO subset in AmiGO database. Normalized enrichment scores (NES) of GO terms are plotted. Source data are provided as a Source Data file.

To further characterize biological responses triggered by SETD2 deletion during PKD-ccRCC transition at the mRNA and protein levels, we next conducted global transcriptomics and proteomics studies. Cysts from KM mice and neoplastic masses from KMS mice were collected for RNA-sequencing and LC-MS/MS proteomics (Fig. 3a). Transcriptomics analysis identified a total of 1072 differential genes (FDR < 0.05, FC > 1.5) (Fig. 3b), including 817 downregulated genes (76.21%) and 255 upregulated genes (23.79%) in KMS mice compared to KM mice (Fig. 3c). Proteomics analysis identified a total of 290 differential proteins (FDR < 0.05, FC > 1.5) (Fig. 3b, Supplementary Fig. 2a), including 160 upregulated proteins (44.83%) and 130 downregulated proteins (55.17%) in KMS mice compared to KM mice (Fig. 3c). Of note, a prominent uncoupling of mRNA and protein expression after SETD2 ablation was revealed through analysis of the differential mRNAs and proteins between KMS and KM (Supplementary Fig. 3a, b).

To further clarify the biological processes and pathways affected by SETD2 deficiency, we implement enrichment analysis based on differential mRNAs and proteins. KEGG and Gene Ontology (GO) enrichment analysis on proteomics data indicated distinct alterations in metabolic pathways (Fig. 3e, Supplementary Fig. 2b, c), which were more significant than that on transcriptomics data (Fig. 3d, Supplementary Fig. 3c). We next integrated proteomic and transcriptomic datasets to determine the dysregulation of cellular processes induced by SETD2 deficiency. By adopting gene set enrichment analysis (GSEA) pathway enrichment, we found half pathways were either upregulated or downregulated at both protein and mRNA levels, including upregulated fatty acid metabolic process and downregulated amino acid metabolism (Fig. 3f). These results demonstrate that SETD2 indeed regulates broad metabolic pathways during PKD-ccRCC transition.

### SETD2 loss promotes metabolic reprogramming and sphingomyelin biosynthesis by regulating metabolic proteins

**Fig. 4:**
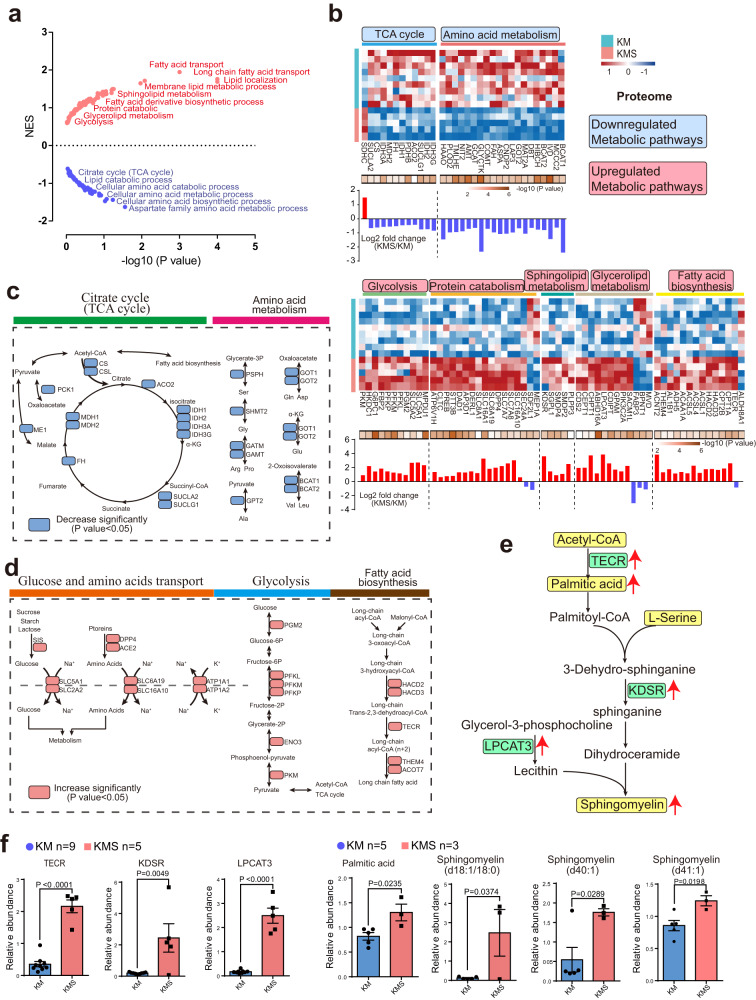
SETD2 deficiency leads to metabolic reprogramming and upregulated sphingomyelin biosynthesis. **a** GSEA of KEGG metabolic pathways enriched in KMS mice compared to KM mice. Upregulated and downregulated pathways are shown in red and blue, respectively. **b** Heat map and quantitative analysis of altered proteins (FDR < 0.05) in upregulated and downregulated pathways between KMS and KM mice. **c, d** Reprogrammed metabolic pathways in KMS mice compared to KM mice. **e** Schema of de novo sphingomyelin biosynthesis with upregulated proteins and metabolites in KMS mice compared to KM mice. The most critical representative proteins and metabolites were highlighted in green and yellow, respectively (FDR < 0.05 and fold change >1.5). **f** KMS mice showed increased TECR (*P* < 0.0001), KDSR (*P* = 0.0049), LPCAT3 (*P* < 0.0001), Palmitic acid (*P* = 0.0235), and Sphingomyelin d18:1/18:0 (*P* = 0.0374), d40:1 (*P* = 0.0299) and d41:1 (*P* = 0.0198) compared to KM mice. Statistical comparisons were made using a 2-tailed Student’s *t* test. Data are represented as mean ± SEM. Source data are provided as a Source Data file.

Given that proteomics analysis clearly indicated the strong connection between metabolic proteins and SETD2 deletion, we further performed GSEA analysis on proteomics dataset to identify the alteration of KEGG metabolic pathways during PKD-ccRCC transition (Fig. 4a). We found that the enriched upregulated pathways in SETD2 deficient renal tissues were frequently related to glycolysis, protein catabolic and lipids transport and metabolism, evidenced by upregulated proteins (FDR < 0.05), such as PKM, CLTC, SLC5A1 and TECR, as well as downregulated pathways including carbohydrate metabolism, TCA cycle and amino acid metabolism, evidenced by downregulated proteins (FDR < 0.05), such as IDH2, CS, and GOT2 (Fig. 4b). The downregulation of TCA cycle (CS, CSL, ACO2, IDH1, IDH2, IDH3A, IDH3G, SUCLA2, SUCLG1, FH, MDH1, MDH2, ME1 and PCK1), amino acid metabolism (PSPH, SHMT2, GATM, GAMT, GPT2, GOT1, GOT2, BCAT1 and BCAT2) components, as well as upregulation of fatty acid biosynthesis (HACD2, HACD3, TECR, THEM4 and ACOT7), carbohydrate absorption (SIS, OPP4, ACE2, SLC5A1, SLC2A2, SLC6A19, SLC16A10, ATP1A1 and ATP1A2), glycolysis (PGM2, PFKL, PFKM, PFKP, ENO3 and PKM) components that we observed at protein level revealed the metabolic reprogramming toward accelerating lipids biosynthesis in SETD2 deficient ccRCC mice (Fig. 4c, d).

In our study, we demonstrated that SETD2 ablation resulted in decreased amino acid metabolism and increased fatty acid biosynthesis during PKD-ccRCC transition. Notably, enhanced levels of amino acid and palmitoyl-CoA are critical to de novo biosynthesis of SM^25^. Our data showed increased protein levels of TECR, KDSR and LPCAT3, which are essential enzymes in SM biosynthesis, as well as increased palmitic acid, sphingomyelin (d18:1/18:0), sphingomyelin (d40:1) and sphingomyelin (d41:1) in KMS samples compared to KM samples (Fig. 4e, f). These results suggest that SETD2-deficiency-induced metabolic alterations contribute to de novo synthesis of SM. Our study reveals a critical function of SETD2 in metabolic reprogramming, while the deletion of SETD2 promotes de novo synthesis of SM during PKD-ccRCC transition.

### SETD2 loss is accompanied by increased sphingomyelin in human ccRCC

**Fig. 5:**
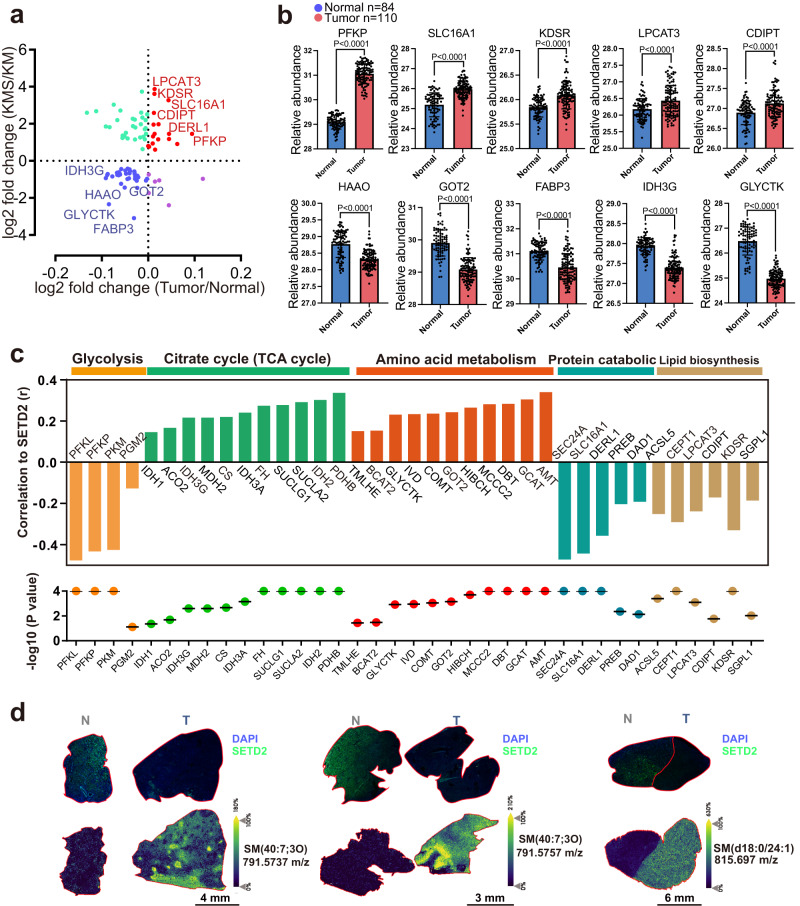
SETD2 loss associates with upregulated sphingomyelin biosynthesis in human ccRCC. **a** Sample-averaged, normalized and log-transformed change level for altered protein pairs in human and mouse ccRCC. Up- and down-regulated proteins at both mouse and human ccRCC are shown in red and blue, respectively. **b** Increased PFKP, SLC16A1, KDSR, LPCAT3 and PFKP proteins, as well as decreased HAAO, GOT2, FABP3, IDH3G and GLYCTK proteins were observed in ccRCC samples compared to adjacent normal kidney tissues (*P* < 0.0001). **c** The correlation of key protein abundances and protein level of SETD2 in human ccRCC. **d** Representative immunofluorescent images showing protein levels of SETD2 in ccRCC patients and representative MALDI-IMS images of ccRCC tissue from the same patients. Statistical comparisons were made using a 2-tailed Student’s *t* test. Data are represented as mean ± SEM. Source data are provided as a Source Data file.

To further examine the clinical functional relevance between SETD2 and SM biosynthesis, we analyzed transcriptomic and proteomic signatures in ccRCC patients from The Cancer Genome Atlas (TCGA) database (*n* = 533) and The Clinical Proteomic Tumor Analysis Consortium (CPTAC) database (*n* = 606), respectively. Clinically, mutations and low expression of SETD2 were widely observed in ccRCC samples (Supplementary Fig. 4a, b). At the protein level, 17 and 38 proteins were upregulated and downregulated respectively at both mouse and human ccRCC, which account for 60.45% of total differential metabolic proteins (Fig. 5a). Specifically, upregulated proteins, including PFKP, SLC16A1, KDSR, LPCAT3 and CDIPT, as well as downregulated proteins, including HAAO, GOT2, FABP3, IDH3G and GLYCTK caused by SETD2 deletion in mice, were also consistently increased or decreased respectively in human ccRCC (Fig. 5b). GSEA analysis on clinical proteomic data from CPTAC database also showed statistically significant enrichment between SETD2 alteration and metabolic pathways (Supplementary Fig. 4c). Correlation analysis showed positive correlations between SETD2 and the levels of proteins in TCA cycle and amino acid metabolism. Meanwhile, the low level of SETD2 was clinically related to the upregulations of proteins in glycolysis, protein catabolic and lipid biosynthesis (Fig. 5c, Supplementary Fig. 4d). These results indicated a positive correlation of SETD2 loss with metabolic reprogramming in human ccRCC, consistent with our previous findings in SETD2 deficient mice.

To further validate the relevance between SETD2 and SM biosynthesis, we detected levels of SETD2 protein and SM in human ccRCC samples. Immunofluorescent (IF) staining showed reduced protein level of SETD2 in ccRCC tissues compared to adjacent normal tissues. Then MALDI imaging mass spectrometry (MALDI-IMS) was performed to measure the in situ metabolites abundance on paired human ccRCC and normal samples with altered SETD2 levels. Our data showed significantly increased SMs in SETD2-low tumors, including SM (40:7;3O, m/z 791.5757), SM (40:6;3O, m/z 793.5889), SM (40:7;3O, m/z 791.5757) and SM (40:5;3O, m/z 795.6071), demonstrating a clear inverse correlation between SETD2 and SM (Fig. 5d and Supplementary Fig. 4e). Collectively, our results demonstrated that SETD2 loss is significantly associated with upregulated SM in human ccRCC.

### Inhibition of sphingomyelin biosynthesis relieves the symptom caused by SETD2 deletion in mice

**Fig. 6:**
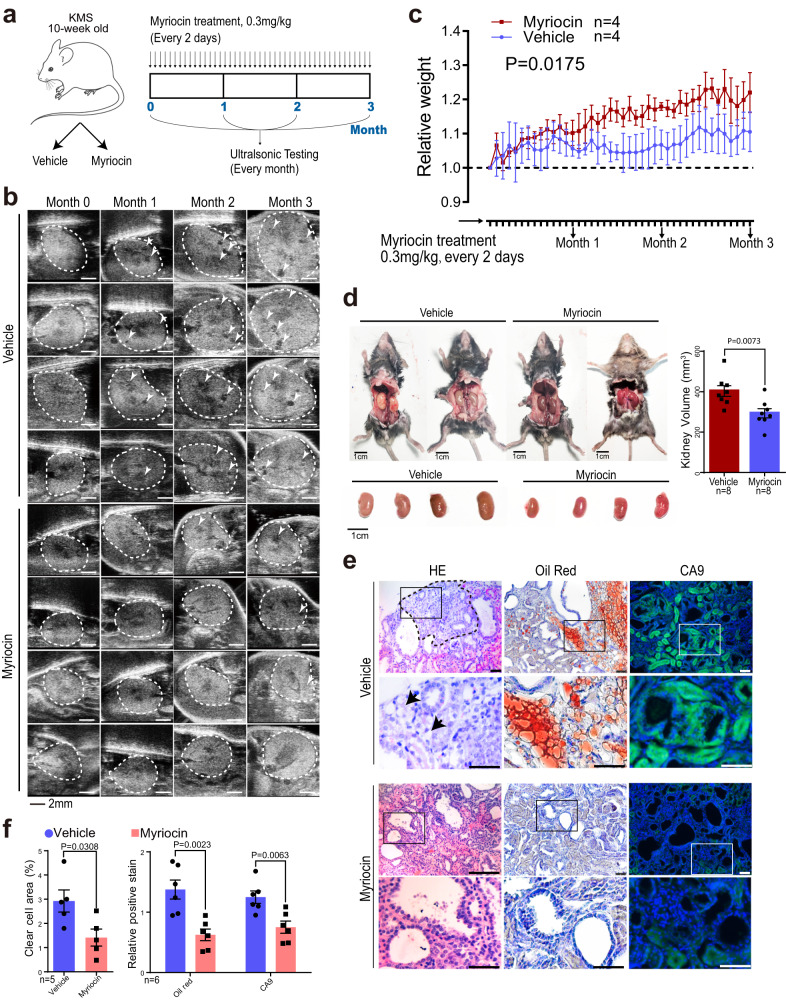
Inhibition of sphingomyelin biosynthesis relieves the tumorigenesis symptom caused by SETD2 deletion in mice. **a** Graphic illustration of the workflow and course of treatment. **b** The longitudinal ultrasonic imaging of tumor development in vehicle- and myriocin-treated KMS mice. Kidneys were indicated by white dotted lines. Scale bars = 2 mm. **c**, **d** Body weights and kidney volumes of vehicle- and myriocin-treated KMS mice. Scale bars =1 cm. Representative images (**e**) and metrics (**f**) showing protein levels and histologic changes upon the treatment of myriocin. Aberrant areas are indicated by black dotted lines and arrows. The area in the box is enlarged on the right. Scale bars, 50 μm. Statistical comparisons were made using a two-tailed Student *t* test. Data are represented as mean ± SEM. Source data are provided as a Source Data file.

In light of the observation that SETD2 deficiency gave rise to enhanced SM content, which is strongly related to cancer initiation, growth, and immune evasion^22^, we were prompted to test whether SM synthesis inhibitors can confer protective effects against SETD2-loss-induced PKD-ccRCC transition in vivo. De novo synthesis of SM is initiated by the condensation of serine and palmitoyl-CoA, which is catalyzed by serine palmitoyl transferase (SPT)^26^. Myriocin, an analog of sphingosine that functions as a potent inhibitor of SPT, has been evaluated in vivo as a metabolic modulator with favorable effects in metabolic syndrome^25^ and cancers^27–29^. In our study, ten-week-old KMS mice were treated with myriocin intraperitoneally (0.3 mg/Kg) every two days for 3 months to block de novo synthesis of SM and subjected to ultrasonic testing every month (Fig. 6a). As depicted in Supplementary Fig. 5a, a significant decrease in SM levels were observed in myriocin-treated KMS mice in comparison to the vehicle-treated KMS mice. Inspiringly, the results of ultrasonic testing before and after treatment showed less neoplastic masses in myriocin-treated mice compared to vehicle-treated mice (Fig. 6b). And Myriocin-treated mice exhibited lighter body weight (*P* = 0.0175) and smaller kidney size (*P* = 0.0073) compared to vehicle-treated mice (Fig. 6b–d). Morphologically, smaller clear-cell areas were observed in myriocin-treated mice compared to vehicle-treated mice. Further histology analysis showed that myriocin-treated mice exhibited attenuated staining of CA9 and less lipid accumulation compared to vehicle-treated mice (Fig. 6e, f). These results intuitively revealed that the ccRCC-related symptoms caused by SETD2 deletion were alleviated and progression from PKD to ccRCC was blocked after the utilization of myriocin. Furthermore, to impede SM synthesis, we also employed lentivirus-mediated knockdown systems targeting Serine Palmitoyltransferase Long Chain Base Subunit 2 (SPTLC2), a pivotal enzyme responsible for initiating the de novo synthesis of SM^26^, in *SETD*-mut ccRCC cell line CAKI-1. Data showed a notable decrease in SM levels (Supplementary Fig. 5b), accompanied by a reduction in cell proliferation both in vitro and in vivo (Supplementary Fig. 5c, d) upon SPTLC2 knockdown. Collectively, the obtained results indicate that SETD2 loss promotes the transition from PKD to ccRCC in a SM dependent manner.

**Fig. 7:**
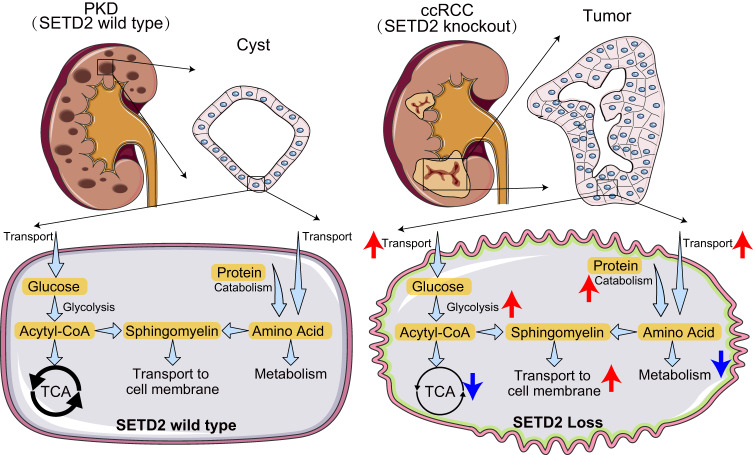
SETD2 deficiency accelerates sphingomyelin accumulation and promotes the transition from PKD to ccRCC. Schematic representing the role of SETD2 deficiency in promoting large-scale metabolic reprogramming and sphingomyelin biosynthesis during PKD-ccRCC transition.

Taken together, our findings demonstrated that SETD2 loss induces extensive and substantial metabolic reprogramming events, including upregulated glycolysis, protein catabolic and fatty acid biosynthesis, as well as downregulated TCA cycle and amino acids metabolism. Metabolic reprogramming caused by SETD2 deletion ultimately accelerates de novo sphingomyelin (SM) synthesis and tumorigenesis during the transition from PKD to ccRCC (Fig. 7).

## Discussion

SETD2 is frequently mutated across cancer types, including lung cancer^30,31^, prostate cancer^32^, intestinal cancer^33^, glioma^34^, gastrointestinal tumors^33,35^, osteosarcoma^36^ and leukemia^37,38^. Genomic analyses on hepatocellular carcinoma^39^ and lung adenocarcinoma^40^ showed that deletions in SETD2 might drive metabolic alterations. Metabolic reprogramming is a hallmark of tumor cells and has been recently regarded as a key contributor in tumor progression^7,41,42^. To better understand the role of the SETD2 in tumor metabolism in RCC progression, we systematically investigated the function of SETD2 in PKD-ccRCC transition based on comprehensive analyses of transcriptomics, proteomics, lipidomics and metabolomics. In our study, the dysregulation of carbohydrate absorption, glycolysis, lipid biosynthesis, sphingolipid metabolic, TCA cycle and amino acid metabolism were observed after *Setd2* knockout. The alterations on these metabolic pathways have been reported to promote tumor growth, progression and metastasis^28,43,44^, and are consistent with the notion that ccRCC cells are lipid- and glycogen-laden^7^. Therefore, our results indicate a strong relation between SETD2 and metabolic reprogramming in ccRCC.

In this study, we identified abnormal biosynthesis of SM in SETD2 deficient ccRCC. Functions of SM on regulating signal transduction have been proposed, such as proliferation/survival, migration and inflammation^22^. These functions are mostly linked to the presence of SM in the plasma membrane, the Golgi or the nucleus^21^. Cell surface biochemical changes, notably excessive increase in outer leaflet SM content, are of importance in cancer initiation, growth and immune evasion^45^. Accumulating evidences suggest that lung, colon, prostate, breast and kidney cancers have relations to SM content and their metabolizing enzymes, confirming that the presence of inordinate SM amount on cancer cells is associated with the level of malignancy^46–48^. Therefore, our work shed light on a mechanism that SETD2 deficiency would exert a crucial impact on SM-mediated biochemical functions and ultimately facilitate the transition from PKD to ccRCC.

A multigene panel that includes PKD1, PKD2, ALG5, ALG9, DNAJB11, GANAB, HNF1B, IFT140 was reported to be relevant to the genetic cause of PKD^49–52^. However, it is worth noting that there is no evidence indicating a common occurrence of SETD2 mutations in PKD, suggesting that SETD2 deficiency have a low relation to PKD process. Meanwhile, mutations of the SETD2 gene are common (occurs up to 12%) in ccRCC patients^10^, thus indicating that SETD2 deficiency may indeed play a crucial role in the development of ccRCC but not PKD. Increased CA9, recognized as a target of VHL/HIF signaling^53,54^, was observed subsequent to *Setd2* knockout in our mouse model. Clinically, low SETD2 is highly related to reduced VHL in mRNA and protein levels (TCGA-KIRC database, Supplementary Fig. 4f, g), suggesting an association between SETD2 loss and decreased VHL expression in the context of ccRCC. This observation provides a foundation for potential future endeavors in basic research, aimed at validating the regulatory influence of SETD2 on VHL/HIF signaling in ccRCC. As a methyltransferase, SETD2-mediated H3K36me3 is essential in ccRCC, and the number of H3K36me3-positive nucleus reduced ~20% in primary ccRCC^10^. In our investigations, altered H3K36me3 modifications on metabolic genes were detected upon SETD2 deficiency (ChIP-seq assays targeting H3K36me3, Supplementary Data 3 and Supplementary Fig. 6a), and 31.87% of altered metabolic proteins exhibited changes in H3K36me3 modifications on their gene bodies (Supplementary Data 4 and Supplementary Fig. 6b). Moreover, SETD2 methyltransferase mutant is deficient on regulating sphingomyelin (Supplementary Fig. 6c). This analysis indicates that SETD2-mediated H3K36me3 modification involves in the alteration of metabolic proteins and sphingomyelin accumulation. Additionally, extant literature have also asserted that SETD2 is able to regulate biological processes through modification of non-histone substrates, such as STAT1^14^ and α-tubulin^15^. It remains to be determined whether SETD2 regulates tumor metabolism by interacting with these proteins and methylating them during PKD-ccRCC transition.

Several ongoing clinical trials have provided preliminary insights into fatty acid and lipid-blocking therapies^55,56^. Of note, in our study, tumor symptom caused by *Setd2* knockout was relieved by myriocin. This finding suggested that SETD2 could be a promising target for metabolic therapy. Myriocin, a selective inhibitor of serine-palmitoyl-transferase (SPT), can block de novo biosynthesis of SM^57,58^. SPT acts as a metabolically responsive “switch” in the lipidome, and inhibition of SPT by myriocin have been co-opted to hinder tumor growth including HCT116, A549 and liver cancer^27,28^. Moreover, contact inhibition, one of the major mechanisms in immune checkpoint underlying the initiation of tumorigenesis, is also affected by the cell surface lipids including SM^45^. Accordingly, SM would be a potential therapeutic target in the anti-tumor immune therapy in the future.

Collectively, we demonstrated that SETD2 deficiency results in large-scale metabolic reprogramming and de novo SM biosynthesis, thereby accelerating the transition from PKD to ccRCC. Our findings reveal the role of the SETD2 in metabolic reprogramming and lipid metabolism in ccRCC. For clinical translation, pharmaceutical investigation of the cross-talks between SETD2 and lipids metabolism especially SM may provide enlightenments for preventing tumorigenesis in PKD patients and ccRCC therapy.

## Methods

### Mouse strains

*Setd2*^*fl/fl*^ mice were generated as previously reported^18^. *MYC*^*R26StopFL/+*^ mice were mated with *Ksp*^*Cre*^ mice to generate *Ksp*^*Cre*^*; MYC*^*R26StopFL/+*^ (*MYC* overexpression) mice in C57BL/6 background. *Setd2*^*fl/fl*^ mice were mated with *Ksp*^*Cre*^*; MYC*^*R26StopFL/+*^ mice to generate *Ksp*^*Cre*^*; MYC*^*R26StopFL/+*^ *Setd2*^*flox/flox*^ (*MYC* overexpression*; Setd2* knockout) mice. *Ksp*^*Cre*^*; MYC*^*R26StopFL/p*^ mice and *Ksp*^*Cre*^*; MYC*^*R26StopFL/p*^*; Setd2*^*flox/flox*^ mice were referred as KM and KMS mice, respectively. All mice were maintained in a specific-pathogen-free (SPF) facility and were housed at a temperature of 25 °C in a humidity-controlled environment with free access to food and water in a 12 h light/dark cycle. All experimental procedures were approved by the Institutional Animal Care and Use Committee of Shanghai Jiao Tong University. Diets for all animal experiments were purchased from SLACOM (Catalogue number: P1101F-25). Our study is not sex- or gender-based, this information has not been collected in our research.

### Histology and immunohistochemistry (IHC) staining

Tissues were fixed in 10% buffered formalin and fixed tissues were sectioned for hematoxylin and eosin (H&E) staining. For IHC staining, paraffin-embedded tissues were deparaffinized, rehydrated, and subjected to a heat-induced epitope retrieval step by treatment with 0.01 M sodium citrate (pH 6.0). Endogenous peroxidase activity was blocked with 0.3% (v/v) hydrogen peroxide in distilled water. The sections were then incubated with 0.3% Triton X-100 in PBS (137 mM NaCl, 2.7 mM KCl, 10 mM Na2HPO4, 2 mM KH2PO4, pH 7.4) for 15 min, followed by 10% goat serum in PBS for 1 hour. Subsequently, samples were incubated with primary antibodies, diluted at appropriate proportion in 1% goat serum overnight at 37 °C. After three washes in PBS, sections were incubated with an HRP-conjugated secondary antibody for 1 h at room temperature. Sections were counterstained with hematoxylin. Three random immunostaining images of each specimen were captured using a Leica DM2500 microscope and analyzed by Image-Pro Plus 6.0 software. Primary antibody against CA9 (NB100-417) was purchased from Novus Biologic. NB anti-CA9 Antibody is a rabbit polyclonal antibody raised against human and mouse CA9 and is recommended for detection of CA9 by IHC at dilution range 1:200 ~ 1:500^59,60^. Anti-rabbit IgG HRP-linked antibody (#7074) was purchased from Cell Signaling Technology.

### Oil-red-O staining and immunofluorescent assays

Tissues were rinsed in ice-cold PBS, fixed in 4% formalin for 15 min at 4 °C, and embedded in Tissue-Tek O.C.T Compound. The sections were stained with freshly prepared Oil-red-O solution for 10 min, then rinsed with 75% ethanol, and nuclei were stained with haematoxylin before examination. For IHC staining, the cover slides were first treated with 0.5% Triton for 10 min and blocked with 10% goat serum at room temperature for 1 h. Primary antibody incubations were performed overnight at 4 °C. After extensive washing with PBS, secondary antibody was applied to the sections at room temperature for 1 h. Slides were washed with PBS three times and then mounted with Vectashield mounting medium (Vector laboratories, Inc. H-1200). Three random immunostaining images of each specimen were captured using a Leica DM2500 microscope and analyzed by Image-Pro Plus 6.0 software. Primary antibody against SETD2 (LS-C332416) was purchased from LifeSpan Biosciences. LS-Bio anti-SETD2 Antibody is a rabbit polyclonal antibody raised against human and mouse SETD2 and is recommended for detection of SETD2 by immunofluorescence at dilution range 1:50 ~ 1:200^39,61^. Primary antibody against CA9 (NB100-417) was purchased from Novus Biologic. NB anti-CA9 Antibody is a rabbit polyclonal antibody raised against human and mouse CA9 and is recommended for detection of CA9 by immunofluorescence at dilution range 1:200 ~ 1:500^59,60^. Anti-rabbit IgG (Alexa Fluor® 488 Conjugate) (#4412) was purchased from Cell Signaling Technology.

### Metabolomics and lipidomics profiling

Fresh kidney tissues from mice were rapidly frozen in liquid nitrogen and then stored intact at −80 °C until LCM analysis. 18-μm thick sections for LCM were thaw-mounted on MembraneSlide (1.0 PEN, ZEISS). ccRCC and PKD tissues were sectioned with a ZEISS PALM system and collected onto AdhesiveCaps (ZEISS).

For metabolomics profiling, the metabolite-containing supernatant was processed and analyzed by Agilent 1290 ultra-high performance liquid chromatography (UPLC) system coupled to a qTOF mass spectrometer (TripleTOF 6600, AB SCIEX). Metabolites separation was performed on an ACQUITY UHPLC BEH Amide column (100 mm × 2.1 mm, 1.7 μm, Waters). A 2 μL supernatant of sample was injected and eluted with a 12 min gradient at a flow rate of 500 μL/min, 40 °C. The LC gradient program was as follows: 0–0.5 min, 95% solvent B (solvent A: 25 mM ammonium acetate and 25 mM ammonium hydroxide in water, solvent B: acetonitrile); 0.5–7 min, 95% to 65% solvent B; 7–8 min, 65% to 40% solvent B; 8–9 min, 40% solvent B; 9–9.1 min, 40–95% solvent B; 9.1–12 min, 95% solvent B. The full-scan mass spectra were acquired in the range of 60-1200 m/z with ESI source settings: temperature, 600 °C; curtain gas, 35 psi; nebulizer gas, 60 psi; heater gas, 60 psi; ion spray voltage, 5000 V for positive-mode detection and −4000 V for negative-mode detection.

For lipidomics profiling, the lipid-metabolite-containing supernatant was processed and analyzed by Agilent 1290 ultra-high performance liquid chromatography (UPLC) system coupled to a qTOF mass spectrometer (TripleTOF 6600, AB SCIEX). Lipid separation was performed on Kinetex-C18 column (100 mm × 2.1 mm, 1.7 μm, Phenomenex). A 2 μL supernatant of sample was injected and eluted with an 18 min gradient at a flow rate of 300 μL/min, 55 °C. The LC gradient program was as follows: 0–12 min, 40–100% solvent B (solvent A: acetonitrile-water (6:4, v/v) with 10 mM ammonium acetate, solvent B: acetonitrile-isopropanol (1:9, v/v) with 10 mM ammonium acetate); 12–14 min, 100% solvent B; 14–14.2 min, 100%-40% solvent B; 14.2–18 min, 40% solvent B. The full-scan mass spectra were acquired in the range of 200–2000 m/z with ESI source settings: temperature, 600 °C; curtain gas, 30 psi; nebulizer gas, 60 psi; heater gas, 60 psi; ion spray voltage, 5000 V for positive-mode detection and −4500V for negative-mode detection. MS raw files were converted by Proteo Wizard and analyzed with XCMS pipeline for peak detection and alignment. The identification of metabolites and lipids were accomplished by MetDNA v1.0 (http://metdna.zhulab.cn/)^62^ and Lipid4DAnalyzer v2.0 (http:// http://lipid4danalyzer.zhulab.cn/)^63^.

### RNA-seq and analyses

Kidney tissue from mice were harvested for RNA preparation. The complementary DNA (cDNA) libraries were prepared using the NEBNext TM Ultra Directional RNA Library Prep Kit, NEBNext Poly (A) mRNA Magnetic Isolation Module, NEBNext Multiplex Oligos according to the manufacturer’s instructions. The products were purified and enriched by PCR to create the final cDNA libraries and quantified by Agilent2200. The tagged cDNA libraries were pooled in equal ratio and used for 150 bp paired-end sequencing in a single lane of the Illumina HiSeqXTen. The raw sequencing data are evaluated by fastp (https://github.com/OpenGene/fastp) including quality distribution of nucleotides, position specific sequencing quality, GC content, the proportion of PCR duplication, kmer frequency etc. These evaluation metrics may help us understand the nature of data deeper before subsequent variant evaluation. Mapping of pair-end reads. Before read mapping, clean reads were obtained from the raw reads by removing the adapter sequences, reads with >5% ambiguous bases (noted as N) and low-quality reads containing more than 20 percent of bases with qualities of <20. The clean reads were then aligned to mouse genome (version: GRCm38 NCBI) using the HISAT2 v2.2.1 (http://daehwankimlab.github.io/hisat2/)^64^. HTseq was used to count gene counts^65^ and RPKM method was used to determine the gene expression. RUVSeq was utilized to eliminate unwanted variation from RNA-Seq data^66^. *P*-value and FDR analysis were subjected to the following criteria: i) Fold Change>1.5 or <0.667; ii) *P*-value < 0.05, FDR < 0.05.

### Proteomics profiling

Tissues were homogenized in RIPA buffer containing protease inhibitor and 100 mM PMSF. Protein lysates were processed by the FASP protocol as described^67^. Proteins were digested with trypsin (Promega) and the peptide solutions were further desalted on a MonoSpin C18 column (GL Sciences). For the proteome library, 80 ug peptides were fractionated using a high pH C18 reverse-phase spin column, the fractionated samples were dried and resuspended for LC-MS/MS measurement.

Sample analysis was performed on an EASY-nLC 1200 system (Thermo Fisher Scientific) coupled to an Orbitrap mass spectrometer (Q Exactive HF-X, Thermo Fisher Scientific). Peptides were loaded to an Acclaim PepMapTM 100 C18 trap column (75 μm × 2 cm, 3 μm, Thermo Fisher Scientific) at 2 µL/min with solvent A (0.1% formic acid in water) and eluted with a 120 min gradient on an Acclaim PepMapTM RSLC C18 analytical column (75 μm × 25 cm, 2 μm, Thermo Fisher Scientific) at a flow rate of 300 nL/min. The gradient elution program was as follows: 0–1 min, 1% to 8% solvent B (acetonitrile-water (8:2, v/v) with 0.1% formic acid); 1–98 min, 8% to 28% solvent B; 98–112 min, 28% to 36% solvent B; 112–116 min, 36%-100% solvent B; 116–120 min, 100% solvent B.

DDA MS parameters were set to: (1) MS: 350–1200 scan range (m/z); 60,000 resolution; 3e6 AGC target; 50 ms maximum injection time (MIT); The 20 most intense ions were fragmented by HCD; (2) HCD-MS/MS: 17 m/z isolation window; 15,000 resolution; 2e5 AGC target; 25 ms MIT; NCE: 28. DIA MS parameters were set to: (1) MS: 350–1200 scan range (m/z); 60,000 resolution; 3e6 AGC target; 50 ms MIT; (2) HCD-MS/MS: 17 m/z isolation window; 30,000 resolution; 5e5 AGC target; automatic MIT; 50 loop count; NCE: 28.

MS raw files were searched against the *Mus musculus* database (UniProt, 2022.01.11), then analyzed in DIA-NN v1.8 (https://github.com/vdemichev/DiaNN/releases/tag/1.8.1)^68^ with default parameters. Up to two missed cleavages were allowed. 1% false discovery rate (FDR) threshold was used in both protein and peptide identifications.

### Chromatin immunoprecipitation sequencing and analyses

Cells were cross-linked with 1% formaldehyde for 10 min at room temperature and quenched with 125 mmol/L glycine. The fragmented chromatin fragments were precleared and then immunoprecipitated with Protein AþG Magnetic beads coupled with anti-H3K36me3 (ab9050) and immunoglobulin G (Santa Cruz Biotechnology). Abcam anti-H3K36me3 antibody (ChIP Grade) is a rabbit polyclonal antibody raised against human and mouse H3K36me3 and is recommended for chromatin immunoprecipitation assay at dilution range 1:200 ~ 1:500^69,70^. After reverse cross-linking, chromatin immunoprecipitation (ChIP) and input DNA fragments were end-repaired and A-tailed using the NEBNext End Repair/dA-Tailing Module (E7442, NEB) followed by adapter ligation with the NEBNext Ultra Ligation Module (E7445, NEB). The DNA libraries were amplified for 15 cycles and sequenced using Illumina NextSeq 500 with single-end 1 × 75 as the sequencing mode. Raw reads were filtered to obtain high-quality clean reads by removing sequencing adapters, short reads (length <50 bp), and low-quality reads using Cutadapt (v1.9.1) and Trimmomatic (v0.35). Then FastQC is used to ensure high reads quality. The clean reads were mapped to the mouse genome (assembly GRCm38) using the Bowtie2 (v2.2.6) software. Peak detection was performed using the MACS (v2.1.1) peak finding algorithm with 0.01 set as the P value cutoff. Annotation of peak sites to gene features was performed using the ChIPseeker and showed in Supplementary Data 3.

### Pathway enrichment analysis

Metabolites with significant differences (VIP > 1) between two groups were used for KEGG enrichment analysis using MetDNA v1.0 (http://metdna.zhulab.cn/)^62^. The GO/KEGG enrichment analysis was executed using clusterProfiler package (version: v.4.5.0)^71^ through Hiplot Pro (https://hiplot.com.cn/), a comprehensive web service for biomedical data analysis and visualization. Gene expression data of mRNA and protein levels were used to obtain NES over the active GO/KEGG biological processes using GSEA software (v.4.1) (http://software.broadinstitute.org/gsea/index.jsp)^72^. GO/KEGG terms were plotted with NES values for mRNA and protein. GO gene sets belonging to Mus musculus used in this article were integrated from AmiGO database (http://amigo.geneontology.org/amigo/landing).

### MALDI imaging mass spectrometry (MALDI-IMS)

Fresh samples of human ccRCC and paired normal tissue used for MALDI-IMS were obtained during surgery at the Ruijin Hospital Affiliated to Shanghai Jiaotong University. All samples were collected with the informed consent of patients and in compliance with the strategy of the Ethics Committees of the Ruijin Hospital, Shanghai Jiaotong University School of Medicine. Our study is not sex- or gender-based, this information has not been collected in our research.

Fresh tissues were rapidly frozen in liquid nitrogen and then sectioned on ITO slides (Bruker Daltonics) with a Cryostat (Thermo Fisher Scientific) at −20 °C. 15 mg/mL DHB (2,5-dihydroxybenzoic acid) matrix solution was sprayed on the tissue surface via the HTX TM-Sprayer (TMSP-M3, HTX Technologies). The slides were imaged on a MALDI-TOF mass spectrometry (timsTOF flex, Bruker Daltonics). Imaging data were acquired in positive ion mode in the range of 300–1500 m/z at 50 μm spatial resolution with 400 laser shots per position. Mass spectra were visualized using SCiLS Lab software (Bruker Daltonics).

### Isolation of primary tubular epithelial cells (PTECs) and cell cultures

The isolation procedures and phenotype identification were performed as previously described^73^. PTECs were maintained in Dulbecco’s modified Eagle’s medium/F-12 GLUTMAX-1 containing 10% fetal bovine serum (FBS), 100 U/ml of penicillin and 100 μg/ml of streptomycin (all used for cell culturing are from Sigma-Aldrich, St. Louis, MO). All the cells were maintained at 37 °C under humidified air containing 5% CO_2_. Mycoplasma, bacteria, and fungi were tested as negative in these cultures.

### SETD2 knockout/inhibition and Sphingomyelin Assay

To investigate the role of SETD2 in sphingomyelins accumulation, lentivirus expressing Cre and SETD2-IN-1 (1 μM) were used to knockout *Setd2* gene and inhibit SETD2 function respectively in renal primary tubular epithelial cells (PTECs) from *Setd2*^*flox/flox*^ mice. Lentiviral packaging plasmids pCMV-DR8.8 and pMD2.G were co-transfected with the backbone plasmid into 293T cells for virus production. Cells were selected in 2.5 µg/mL puromycin in the culture medium or by fluorescence-activated cell sorting to generate the stable transfections. PTECs were then cultured and harvested for sphingomyelin assay. Sphingomyelin Quantification Colorimetric Assay Kit (ab287856) was used for measuring the levels of sphingomyelins in PTECs.

### Plasmids, transfection, and lentivirus

h-SETD2 methyltransferase mutant (c.4871C>G, p.S1624C^74^) plasmid was a kind gift from Guan Ning Lin (Shanghai Jiao Tong University, Shanghai, China). Human SETE2 cDNA was generated by polymerase chain reaction. The cDNAs of p.S1624C SETD2 and wild-type SETD2 was cloned into pCMV6-Entry vector with HA-tag. ShRNA sequences for SPTLC2 and scramble shRNA were cloned into lentiviral vector pLKO.1-GFP-puro (sh-SPTLC2#1: CTTCGAGATTTCTTGAGGTAT; sh-SPTLC2#2 GCTCATACCAAAGAAATACTT). For transfection, cells were transfected with the jetPRIME transfection reagent (Polyplus) according to the manufacturer’s instruction. All the constructs generated were confirmed by DNA sequencing. Lentiviral packaging plasmids pCMV-DR8.8 and pMD2.G were co-transfected with the backbone plasmid into 293 T cells for virus production. Cells were selected in 2 µg/mL puromycin in the culture medium to generate the stable transfections.

### In vitro proliferation assays

CAKI-1 cell line were obtained from the American Type Culture Collection (ATCC) and were confirmed by specific indices. CAKI-1 cells were cultured in RPMI-1640 supplemented with 10% FBS (Thermo), 100 U/mL penicillin, and 0.1 mg/mL streptomycin (Thermo) at 37 °C in humidified 5% CO2 atmosphere. sh-SPTLC2#1 and control CAKI-1 cells were plated in 96-well plates and examined at 24, 48 and 72 h after plating (*n* = 3). Cells were incubated with CellTiter 96 AQueous (MTS) solution for 2 h. The absorbance at 490 nm was then measured using a microplate reader (BioTek).”

### In vivo xenograft assay

Cell suspensions (1 × 10^6^ cells) of sh-SPTLC2#1 and control CAKI-1 cells, in a total volume of 100 μL mixed with matrigel in a 1:1 ratio, were injected subcutaneously into the right flanks of five 4-week-old male BALB/C nude mice (SLAC, Shanghai) respectively. Tumor volume was calculated with the following formula: volume = 4/3 × π × tumor length/2 × tumor width/2^2^. Tumors were collected and photographed 20 days after inoculation. All mice were maintained in a specific-pathogen-free (SPF) facility and were housed at a temperature of 25 °C in a humidity-controlled environment with free access to food and water in a 12 h light/dark cycle. Diets for all animal experiments were purchased from SLACOM (Catalogue number: P1101F-25). All animal experiments were carried out following the ethical regulations of the Institutional Animal Care and Use Committee of Shanghai Jiao Tong University. The maximal tumor size of 1500 mm^3^ was permitted by the Animal Care Committee. In our study, the maximal tumor size was not exceeded.

### Quantification and statistical analysis

Statistical evaluation was conducted using Student’s *t* test. Multiple comparisons were analyzed first by one-way analysis of variance. The Pearson correlation was used to analyze the strength of the association between expression levels of SETD2 and its related proteins in patient samples. A significant difference was defined as *P* < 0.05.

### Statistical analysis

Differences between two groups were assessed using two-tailed unpaired Student’s *t* tests. Multiple comparisons were analyzed first by one-way analysis of variance. The Pearson correlation was used to analyze the strength of the association between expression levels of SETD2 and its related genes in patient samples. A significant difference was defined as *P* < 0.05. GraphPad Prism v8.4.2.679 was used for all statistical analyses.

### Reporting summary

Further information on research design is available in the Nature Portfolio Reporting Summary linked to this article.

### Supplementary information

Supplementary Information

Description of Additional Supplementary Files

Supplementary Data 1

Supplementary Data 2

Supplementary Data 3

Supplementary Data 4

Reporting Summary

### Source data

Source Data

## Data Availability

The gene expression data for renal clear cell carcinoma (KIRC) was downloaded from The Cancer Genome Atlas database (TCGA, https://www.genome.gov/Funded-Programs-Projects/Cancer-Genome-Atlas), which were processed by Broad Institute’s TCGA workgroup. The RNA-seq level 3 gene expression data contain log2- or log10-transformed RNA-seq by expectation maximization (RSEM) values summarized at gene level. The proteomic signature for renal clear cell carcinoma was downloaded from The Clinical Proteomic Tumor Analysis Consortium database (CPTAC, Study ID: PDC000127). Our study is not sex- or gender-based, this information has not been collected in our research. Transcriptomics (RNA-seq) and ChIP-seq raw data have been deposited in the Gene Expression Omnibus (GEO) under accession number GEO: GSE221136 and GSE125528. Proteomics raw data have been deposited in the ProteomeXchange Consortium via the PRIDE^75^ partner repository with dataset identifier PXD038966. Proteomics raw files were searched against the *Mus musculus* database (UniProt, https://www.uniprot.org/, 2022.01.11). Raw metabolomics and lipidomics data are included in Supplementary Data 1 and Supplementary Data 2 and are available at https://github.com/Rao-HY/Omics-data-of-SETD2-Deficient-Cell-Renal-Cell-Carcinoma. The spectrum of lipids was categorized via the ClassyFire classification database (http://classyfire.wishartlab.com). Source data are provided with this paper. GraphPad Prism software v8.4.2.679 was utilized to visualize the data in the form of dot, pie, or bar charts. Adobe Illustrator software was utilized to display metabolic and cartoon schemas. Heatmaps were constructed using TBtools (version 1.0986; https://github.com/CJ-Chen/TBtools/releases)^76^. Source data are provided with this paper.

## Code availability

The processing and visualization of data in our manuscript were executed through online bioinformatics platforms as well as stand-alone software applications as described in our manuscript. Codes for data analysis are available at https://github.com/Rao-HY/Omics-data-of-SETD2-Deficient-Cell-Renal-Cell-Carcinoma. 10.5281/zenodo.8416204.
