# Supplementary material for: SETD2 deficiency accelerates sphingomyelin accumulation and promotes the development of renal cancer
Source: Nat Commun. 2023 Nov 21;14:7572. doi: 10.1038/s41467-023-43378-w (PMC10663509; doi:10.1038/s41467-023-43378-w)
Supplement: Supplementary file 1 — Supplementary Information [file 41467_2023_43378_MOESM1_ESM.pdf]

SETD2 Deficiency Accelerates Sphingomyelin Accumulation and Promotes the Development of Renal Cancer

Supplementary Figures

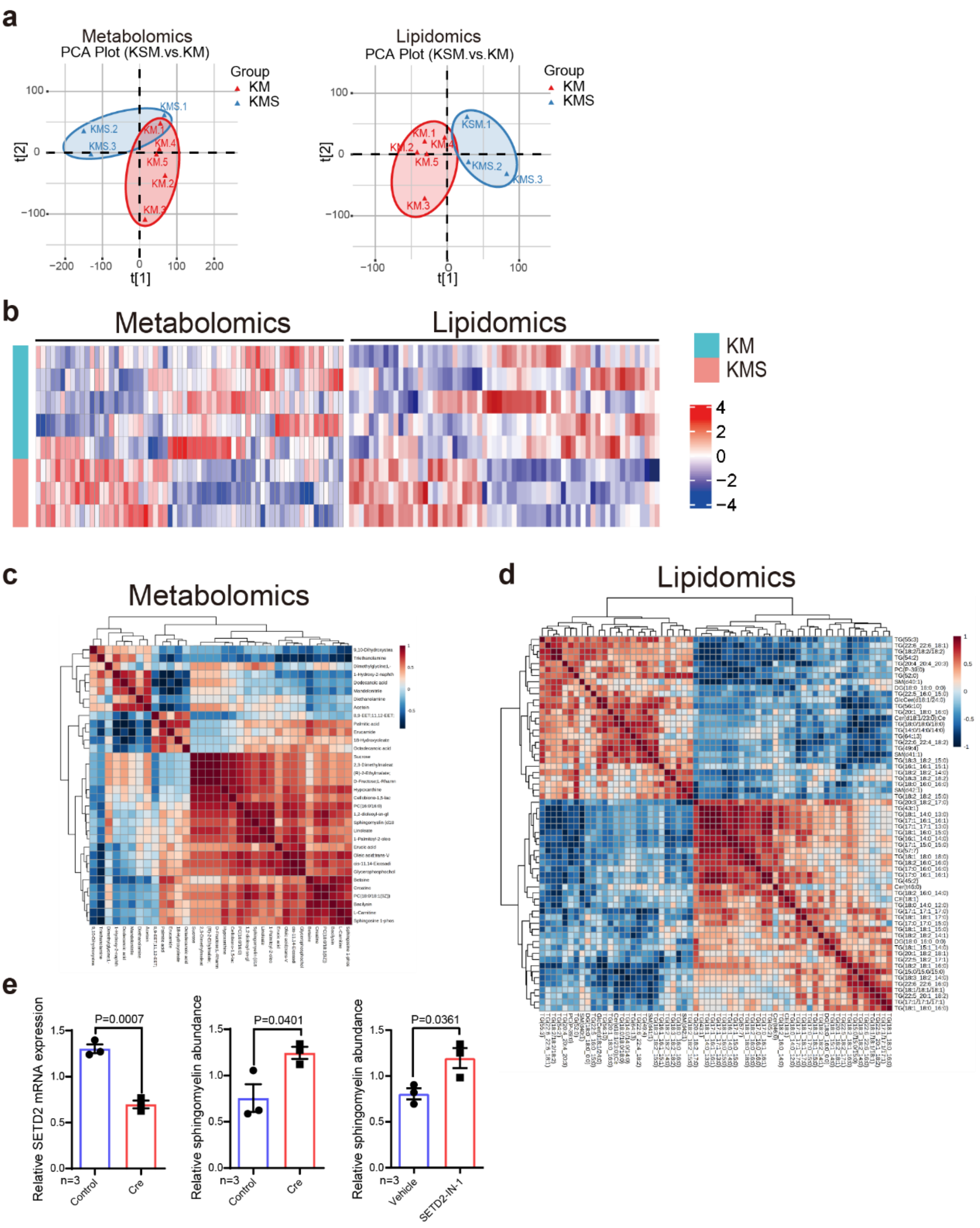

Supplementary Fig. 1. Metabolomics and lipidomics analyses of SETD2 deficient ccRCC. a Principal component analysis for the detected metabolites and lipids in kidneys form KM and KMS mice. **b** Heat map of the altered metabolites and lipids between KM (n=5) and KMS (n=3) mice. Pearson correlations of metabolomics (**c**) and lipidomics (**d**) data between kidneys from KM and KMS mice. **e** Relative sphingomyelin abundances in renal tubular epithelial cells after SETD2 knockout and SETD2-IN-1 treatment. Statistical

comparisons were made using a two-tailed Student t test. Data are represented as mean ± SEM. Source data are provided as a Source Data file.

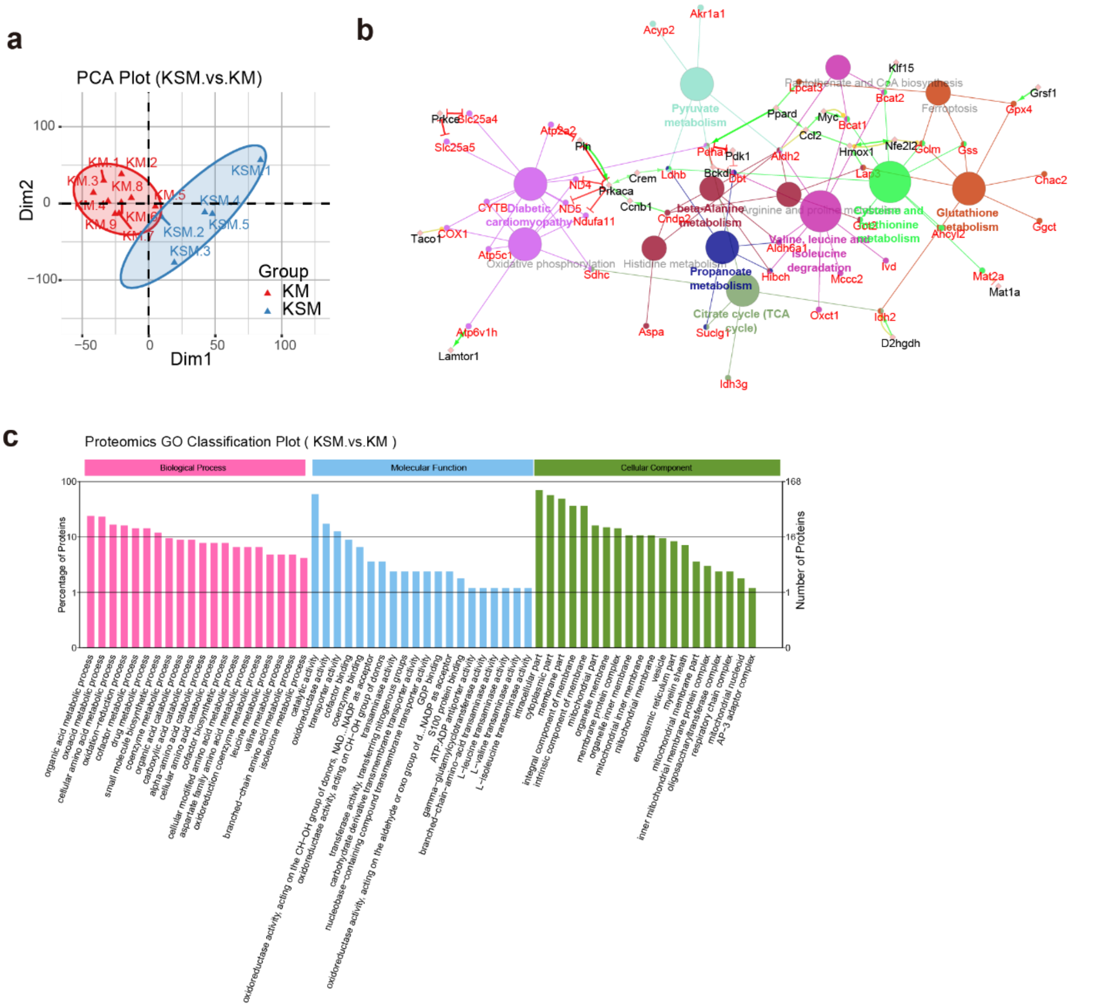

Supplementary Fig. 2. Proteomics analysis of SETD2 deficient ccRCC. a Principal component analysis for the detected proteins in kidneys form KM and KMS mice. **b** The PPI network diagram depicting the interaction of the metabolic pathways. **c** Gene Ontology (GO) classification of differential proteins in KMS kidneys compared to KM kidneys. Source data are provided as a Source Data file.

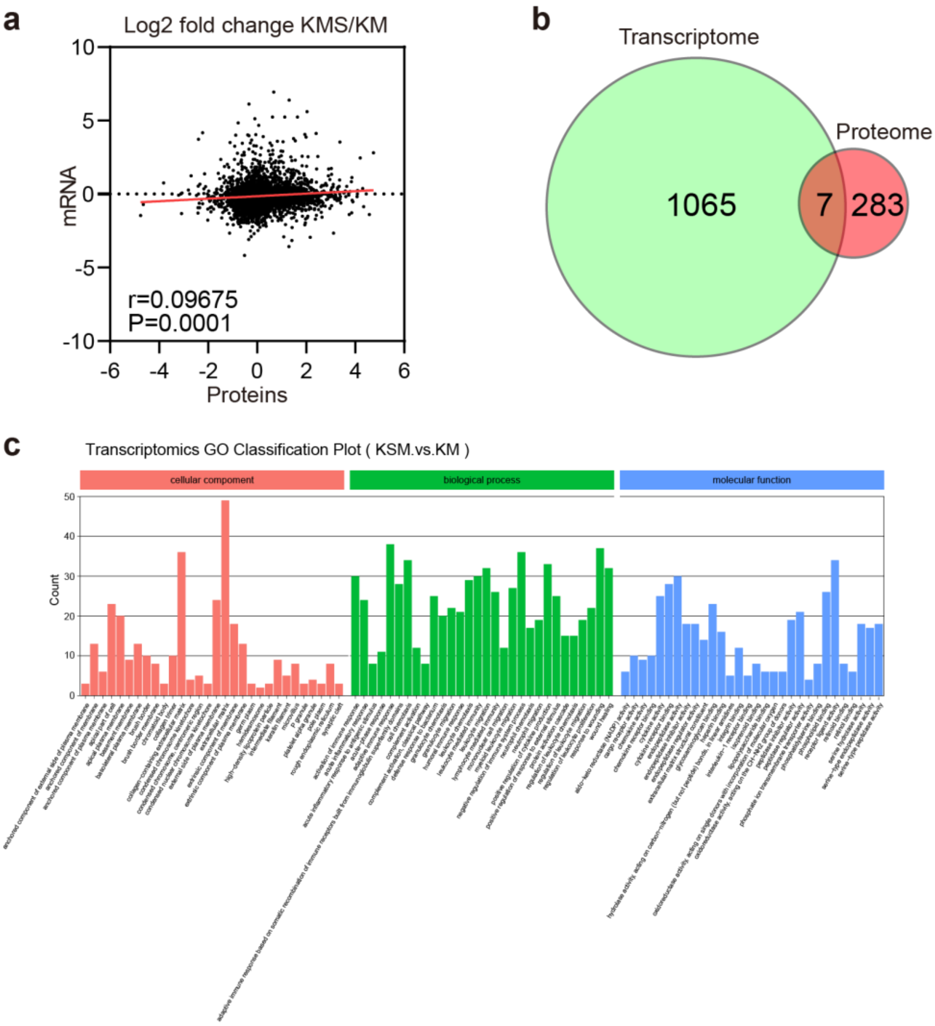

Supplementary Fig. 3. Uncoupling of mRNA and protein expressions in SETD2 deficient ccRCC. a Scatterplots depicting the expressions of protein (x axis) and mRNA (y axis). Linear regression of all mRNA-protein pairs (red line) is shown. **b** Overlap of differential proteins (proteome) and differential genes (transcriptome) between KMS and KM kidneys. **c** Gene Ontology (GO) classification of differential genes between KMS and KM kidneys. Source data are provided as a Source Data file.

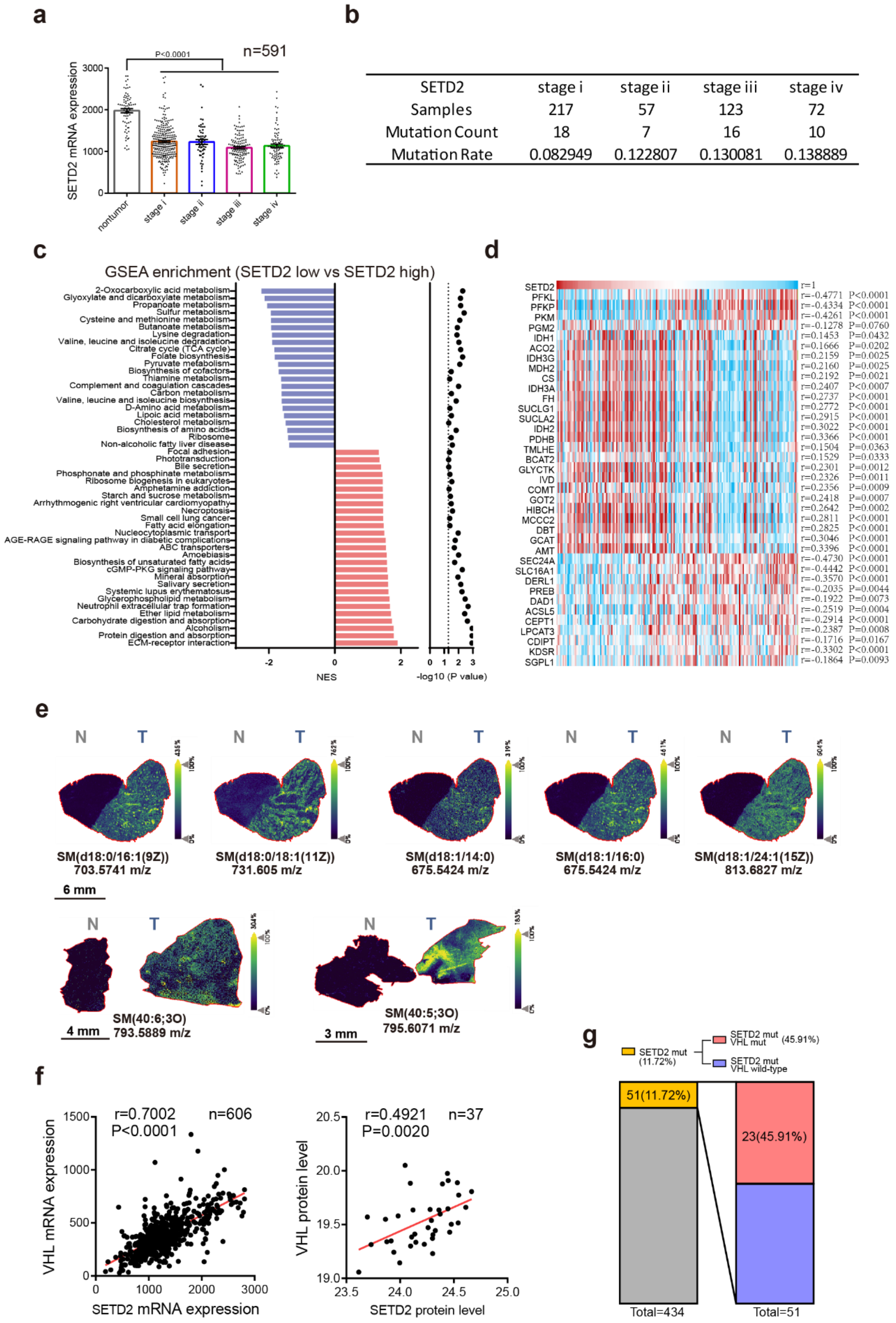

Supplementary Fig. 4. SETD2 deficiency is highly relevant to metabolism in ccRCC. **a** Expression levels of SETD2 in ccRCC stages. **b** Mutation rates of SETD2 in ccRCC stages. **c** GSEA classification of differential proteins in human SETD2-low ccRCC samples compared to ETD2-high ccRCC samples. **d** The correlation of key protein abundances and

protein level of SETD2 in human ccRCC. **e** Representative MALDI-IMS images of paired ccRCC tissue. **f** Correlations between the mRNA and protein levels of SETD2 and VHL in ccRCC samples. **g** Half SETD2-mut ccRCC samples were harboring VHL mutation. Statistical comparisons were made using a two-tailed Student t test. Data are represented as mean ± SEM. The Pearson correlation was used to analyze the strength of the association between expression levels of SETD2 and its related genes in patient samples. Source data are provided as a Source Data file.

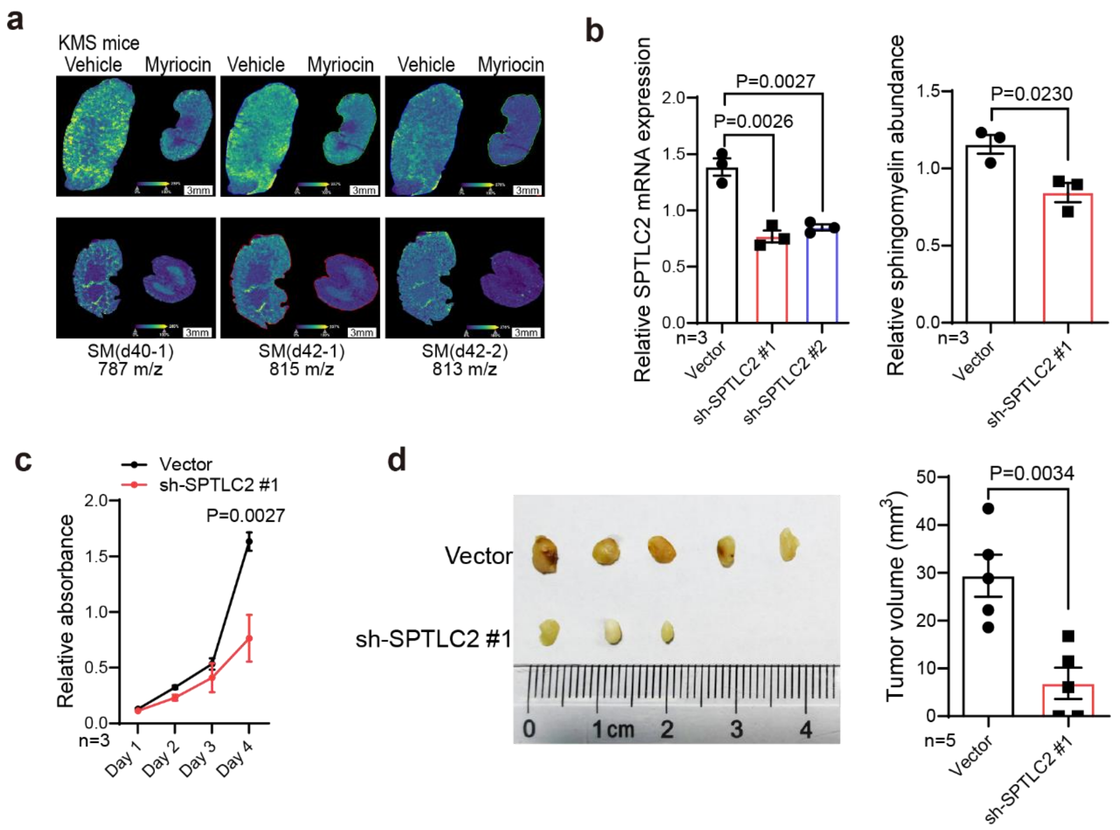

Supplementary Fig. 5. Inhibition of sphingomyelin biosynthesis attenuate cell proliferation. a Representative MALDI-IMS images of kidneys from KMS mice. **b** Relative mRNA level of SPTLC2 and sphingomyelin abundances after SPTLC2 knockdown in CAKI-1 cells. **c** Cell proliferation abilities after SPTLC2 knockdown were compared with their vector control cells. **d** Xenograft tumor assays using CAKI-1 cells stably transfected by SPTLC2 knockdown lentiviruses. Statistical comparisons were made using a two-tailed Student t test. Data are represented as mean ± SEM. Source data are provided as a Source Data file.

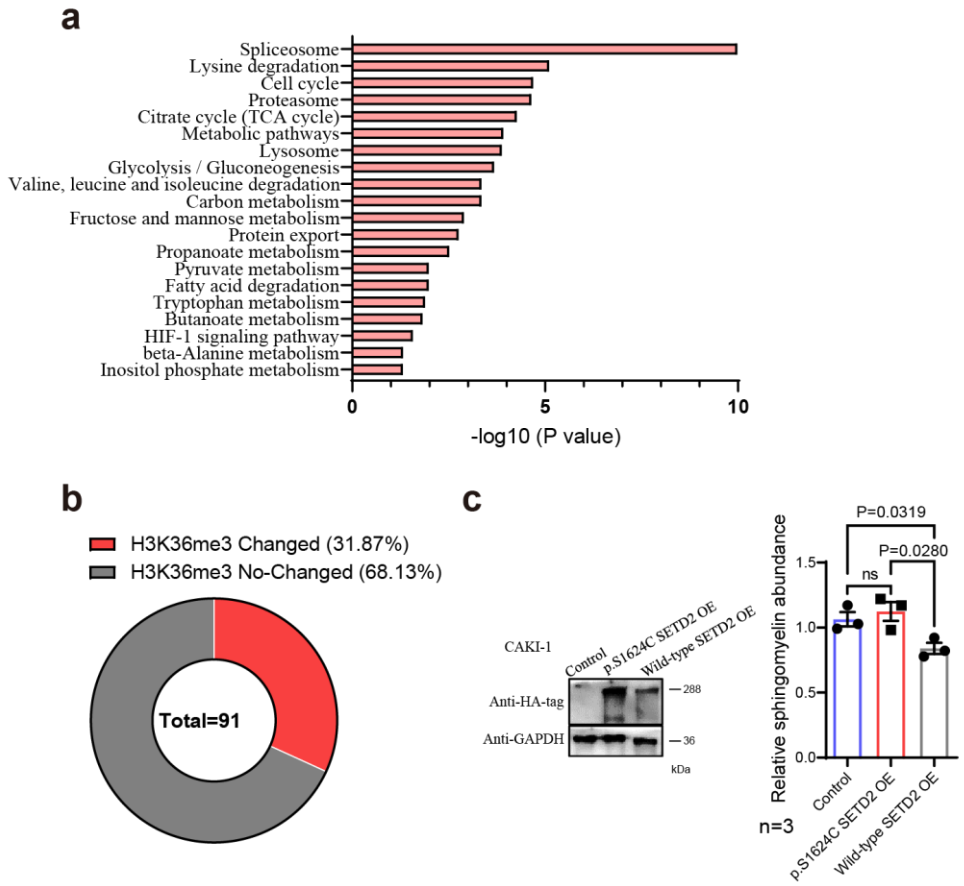

Supplementary Fig. 6. Methyltransferase activity is important for SETD2 to regulate metabolic processes. a KEGG enrichment of H3K36me3 modified genes. **b** 29 of 91 altered metabolic proteins described in Fig. 4b displayed changes in H3K36me3 modifications on their gene bodies. **c** Relative sphingomyelin abundances in CAKI-1 cells after p.S1624C SETD2 and wild-type SETD2 overexpression. Statistical comparisons were made using a two-tailed Student t test. Data are represented as mean ± SEM. Source data are provided as a Source Data file.

Supplementary Data

Supplementary Data 1. Raw mass spectrometry data of metabolomics from KMS and KM kidneys.

Supplementary Data 2. Raw mass spectrometry data of lipidomics from KMS and KM kidneys.

Supplementary Data 3. DNA sites that are modified by HEK36me3.

Supplementary Data 4. Different-expressed metabolic proteins with altered H3K36me3 modification.

Source Data

Source Data for the Supplementary Figures
