# Supplementary material for: SETD2 deficiency accelerates sphingomyelin accumulation and promotes the development of renal cancer
Source: Nat Commun. 2023 Nov 21;14:7572. doi: 10.1038/s41467-023-43378-w (PMC10663509; doi:10.1038/s41467-023-43378-w)
Supplement: Supplementary file 2 — Description of Additional Supplementary Files [file 41467_2023_43378_MOESM2_ESM.pdf]

Description of Additional Supplementary Files

Supplementary Data 1. Raw mass spectrometry data of metabolomics from KMS and KM kidneys.

Supplementary Data 2. Raw mass spectrometry data of lipidomics from KMS and KM kidneys.

Supplementary Data 3. DNA sites that are modified by HEK36me3.

Supplementary Data 4. Different-expressed metabolic proteins with altered H3K36me3 modification.
