# Supplementary material for: SETD2 deficiency accelerates sphingomyelin accumulation and promotes the development of renal cancer
Source: Nat Commun. 2023 Nov 21;14:7572. doi: 10.1038/s41467-023-43378-w (PMC10663509; doi:10.1038/s41467-023-43378-w)
Supplement: Supplementary file 7 — Reporting Summary [file 41467_2023_43378_MOESM7_ESM.pdf]

nature portfolio

Corresponding author(s): Xianting Ding
Last updated by author(s): Oct 8, 2023

Reporting Summary

Nature Portfolio wishes to improve the reproducibility of the work that we publish. This form provides structure for consistency and transparency in reporting. For further information on Nature Portfolio policies, see our Editorial Policies and the Editorial Policy Checklist.

Statistics

For all statistical analyses, confirm that the following items are present in the figure legend, table legend, main text, or Methods section.

n/a	Confirmed	
☐	☒	The exact sample size (*n*) for each experimental group/condition, given as a discrete number and unit of measurement
☐	☒	A statement on whether measurements were taken from distinct samples or whether the same sample was measured repeatedly
☐	☒	The statistical test(s) used AND whether they are one- or two-sided<br>*Only common tests should be described solely by name; describe more complex techniques in the Methods section.*
☐	☒	A description of all covariates tested
☐	☒	A description of any assumptions or corrections, such as tests of normality and adjustment for multiple comparisons
☐	☒	A full description of the statistical parameters including central tendency (e.g. means) or other basic estimates (e.g. regression coefficient) AND variation (e.g. standard deviation) or associated estimates of uncertainty (e.g. confidence intervals)
☐	☒	For null hypothesis testing, the test statistic (e.g. *F*, *t*, *r*) with confidence intervals, effect sizes, degrees of freedom and *P* value noted<br>*Give P values as exact values whenever suitable.*
☐	☒	For Bayesian analysis, information on the choice of priors and Markov chain Monte Carlo settings
☐	☒	For hierarchical and complex designs, identification of the appropriate level for tests and full reporting of outcomes
☐	☒	Estimates of effect sizes (e.g. Cohen's *d*, Pearson's *r*), indicating how they were calculated

Our web collection on statistics for biologists contains articles on many of the points above.

Software and code

Policy information about availability of computer code

Data collection	The identification of metabolites and lipids were accomplished by MetDNA v1.0 (http://metdna.zhulab.cn/) and Lipid4DAnalyzer v2.0 (http://lipid4danalyzer.zhulab.cn/). The spectrum of lipids was categorized via the ClassyFire classification system (http://classyfire.wishartlab.com). The sequencing reads were mapped to the mouse genome (assembly GRCm38) using the HISAT2 software v2.2.1 (http://daehwankimlab.github.io/hisat2/). Proteomics raw files were searched against the Mus musculus database (UniProt, https://www.uniprot.org/, 2022.01.11), then analyzed in DIA-NN v1.8 (https://github.com/vdemichev/DiaNN/) with default parameters.
Data analysis	The processing and visualization of data in our manuscript were executed through online bioinformatics platforms as well as stand-alone software applications as described in our manuscript. KEGG enrichment analysis for metabolites was accomplished by MetaboAnalyst v.4.0 (http://www.metaboanalyst.ca/). The gene GO/KEGG enrichment analysis was executed using clusterProfiler package v.4.5.0 through Hiplot Pro (https://hiplot.com.cn/). GSEA software v.4.1 (http://software.broadinstitute.org/gsea/index.jsp) was used to obtain NE score (NES). GO gene sets belonging to Mus musculus used in this article were integrated from AmiGO database (http://amigo.geneontology.org/amigo/landing).Graphpad Prism software v8.4.2.679 was utilized to visualize the data in the form of dot, pie, or bar charts. Adobe Illustrator software was utilized to display metabolic and cartoon schemas. Heatmaps were constructed using TBtools (version 1.0986; https://github.com/CJ-Chen/TBtools/releases). Codes for data analysis are available at https://github.com/Rao-HY/Omics-data-of-SETD2-Deficient-Cell-Renal-Cell-Carcinoma. DOI: 10.5281/zenodo.8416204.

For manuscripts utilizing custom algorithms or software that are central to the research but not yet described in published literature, software must be made available to editors and reviewers. We strongly encourage code deposition in a community repository (e.g. GitHub). See the Nature Portfolio guidelines for submitting code & software for further information.

Data

Policy information about availability of data

All manuscripts must include a data availability statement. This statement should provide the following information, where applicable:

- Accession codes, unique identifiers, or web links for publicly available datasets
- A description of any restrictions on data availability
- For clinical datasets or third party data, please ensure that the statement adheres to our policy

The gene expression data for renal clear cell carcinoma (KIRC) was downloaded from The Cancer Genome Atlas database (TCGA, https://www.genome.gov/Funded-Programs-Projects/Cancer-Genome-Atlas), which were processed by Broad Institute's TCGA workgroup. The RNA-seq level 3 gene expression data contain log2- or log10-transformed RNA-seq by expectation maximization (RSEM) values summarized at gene level. The proteomic signature for renal clear cell carcinoma was downloaded from The Clinical Proteomic Tumor Analysis Consortium database (CPTAC, Study ID: PDC000127, https://proteomic.datacommons.cancer.gov/pdc/study/PDC000127). Our study is not sex- or gender-based, this information has not been collected in our research.

The datasets involved in the current study are available from the corresponding author on reasonable request. Transcriptomics (RNA-seq) and ChIP-seq raw data have been deposited in the Gene Expression Omnibus (GEO) under accession number GEO: GSE221136 (https://www.ncbi.nlm.nih.gov/geo/query/acc.cgi?acc=GSE221136) and GSE125528 (https://www.ncbi.nlm.nih.gov/geo/query/acc.cgi?acc=GSE125528). Proteomics raw data have been deposited in the ProteomeXchange Consortium via the PRIDE partner repository with dataset identifier PXD038966 (https://proteomecentral.proteomexchange.org/cgi/GetDataset?ID=PXD038966; Username: reviewer_pxd038966@ebi.ac.uk; Password: SScWMID2). Proteomics raw files were searched against the Mus musculus database (UniProt, https://www.uniprot.org/, 2022.01.11). Raw metabolomics and lipidomics data are included in Supplementary Table 1 and Supplementary Table 2 and are available at https://github.com/Rao-HY/Omics-data-of-SETD2-Deficient-Cell-Renal-Cell-Carcinoma. The spectrum of lipids was categorized via the ClassyFire classification database (http://classyfire.wishartlab.com). Source data are provided with this paper.

Research involving human participants, their data, or biological material

Policy information about studies with human participants or human data. See also policy information about sex, gender (identity/presentation), and sexual orientation and race, ethnicity and racism.

Reporting on sex and gender	Our study is not sex- or gender-based, this information has not been collected in our research.
Reporting on race, ethnicity, or other socially relevant groupings	Our study do not involve race, ethnicity, or other socially relevant groupings.
Population characteristics	Samples used in our study were obtained from Chinese ccRCC patients. Our study do not involve age, genotypic information, past and current diagnosis and treatment categories.
Recruitment	Fresh samples of human ccRCC and paired normal tissue were obtained during surgery at the Ruijin Hospital Affiliated to Shanghai Jiaotong University. In this paper, patients were randomly recruited without self-selection bias.
Ethics oversight	All samples were collected with the informed consent of patients and in compliance with the strategy of the Ethics Committees of the Ruijin Hospital, Shanghai Jiaotong University School of Medicine.

Note that full information on the approval of the study protocol must also be provided in the manuscript.

Field-specific reporting

Please select the one below that is the best fit for your research. If you are not sure, read the appropriate sections before making your selection.

[x] Life sciences [ ] Behavioural & social sciences [ ] Ecological, evolutionary & environmental sciences

For a reference copy of the document with all sections, see nature.com/documents/nr-reporting-summary-flat.pdf

Life sciences study design

All studies must disclose on these points even when the disclosure is negative.

Sample size	In our study, we ensure statistical validity by maintaining a minimum sample size of at least three in each group, no sample size calculation was performed. Five KM mice and five KMS mice were used for mouse phenotype study. Kidney cysts from five KM mice and neoplastic masses from three KMS mice were isolated for metabolomic and lipidomic analyses. Kidney cysts from five KM mice and neoplastic masses from five KMS mice were isolated for mRNA-seq analyses. Kidney cysts from nine KM mice and neoplastic masses from five KMS mice were isolated for proteomic analysis. When conducting clinical data inalysis, we prioritize selecting databases with larger sample sizes to derive more comprehensive and generalized conclusions.
Data exclusions	No data were excluded from the analyses.
Replication	In the mouse phenotype study, we performed at least three replicates to ensure the reliability of the results. In multi-omics studies involving mouse experiments, at least three samples per group were tested to ensure statistical validity and no replications were performed. For clinical data analysis, we analysed data from multiple sources and ultimately chose the database with the larger sample size.
Randomization	Mouse samples were allocated according to their genotype. Human samples were allocated according to tumor or normal tissue.

Blinding | Investigators were blinded to group allocation.

Reporting for specific materials, systems and methods

We require information from authors about some types of materials, experimental systems and methods used in many studies. Here, indicate whether each material, system or method listed is relevant to your study. If you are not sure if a list item applies to your research, read the appropriate section before selecting a response.

Materials & experimental systems

n/a	Involved in the study
☐	☒ Antibodies
☐	☒ Eukaryotic cell lines
☒	☐ Palaeontology and archaeology
☐	☒ Animals and other organisms
☒	☐ Clinical data
☒	☐ Dual use research of concern
☒	☐ Plants

Methods

n/a	Involved in the study
☐	☒ ChIP-seq
☒	☐ Flow cytometry
☒	☐ MRI-based neuroimaging

Antibodies

Antibodies used: Primary antibody against SETD2 (LS-C332416) was purchased from LifeSpan Biosciences. Primary antibody against CA9 (NB100-417) was purchased from Novus Biologic. Primary antibody against H3K36me3 (ab9050) was purchased from abcam. Anti-rabbit IgG HRP-linked antibody (#7074) and anti-rabbit IgG antibody (Alexa Fluor® 488 Conjugate) (#4412) were purchased from Cell Signaling Technology.

Validation: LS-Bio anti-SETD2 Antibody is a rabbit polyclonal antibody raised against human and mouse SETD2 and is recommended for detection of SETD2 by immunofluorescence at dilution range 1:50~1:200. (Li, Xue-Jing et al. Hepatology, 2021; Xu, Lichao et al. Science advances, 2021)
NB anti-CA9 Antibody is a rabbit polyclonal antibody raised against human and mouse CA9 and is recommended for detection of CA9 by immunohistochemistry at dilution range 1:200~1:500 and immunofluorescence at dilution range 1:200~1:500. (Becker, Lisa M et al. Cell reports, 2020; Chen, Shiyou et al. Science advances, 2020)
Abcam anti-H3K36me3 antibody (ChIP Grade) is a rabbit polyclonal antibody raised against human and mouse H3K36me3 and is recommended for chromatin immunoprecipitation assay at dilution range 1:200~1:500. (Stewart-Morgan, Kathleen R et al. Nature cell biology, 2023; Barral, Amandine et al. Molecular cell, 2022)

Eukaryotic cell lines

Policy information about cell lines and Sex and Gender in Research

Cell line source(s): CAKI-1 cell line were obtained from the American Type Culture Collection (ATCC) and were confirmed by specific indices.

Authentication: CAKI-1 is an epithelial cell line with SETD2 loss and VHL WT. In our study, we authenticated CAKI-1 cell line by morphological assays, as well as SETD2 and VHL expression assays.

Mycoplasma contamination: All cell lines tested negative for mycoplasma.

Commonly misidentified lines (See ICLAC register): Our research do not involve misidentified cell lines.

Animals and other research organisms

Policy information about studies involving animals; ARRIVE guidelines recommended for reporting animal research, and Sex and Gender in Research

Laboratory animals: Twenty-week-old male and female C57BL/6 mice were used for phenotyping and multi-omics analyses. Ten-week-old male and female C57BL/6 mice were treated with myriocin and vehicle control. Four-week-old male BALB/C nude mice were used for xenograft assay. All mice were maintained in a specific-pathogen-free (SPF) facility and were housed at a temperature of 25 °C in a humidity-controlled environment with free access to food and water in a 12 h light/dark cycle.

Wild animals: The study did not involve wild animals.

Reporting on sex: Our study is not sex- or gender-based, this information has not been collected in our research.

Field-collected samples: The study did not involve samples collected from the field.

Ethics oversight: All mice were maintained in a specific-pathogen-free (SPF) facility and all experimental procedures were approved by the institutional

biomedical research ethics committee at School of Biomedical Engineering, Shanghai Jiao Tong University.

Note that full information on the approval of the study protocol must also be provided in the manuscript.

Plants

Seed stocks	NA
Novel plant genotypes	NA
Authentication	NA

ChIP-seq

Data deposition

☒ Confirm that both raw and final processed data have been deposited in a public database such as GEO.

☒ Confirm that you have deposited or provided access to graph files (e.g. BED files) for the called peaks.

Data access links
May remain private before publication.

ChIP-seq raw data has been deposited in the Gene Expression Omnibus (GEO) under accession number GSE125528 (https://www.ncbi.nlm.nih.gov/geo/query/acc.cgi?acc=GSE125528).

Files in database submission

annoTable_S17vsINPUT_S19_peaks.xls
annoTable_S16vsINPUT_S18_peaks.xls
S17vsINPUT_S19_peaks.loc.xls
S16vsINPUT_S18_peaks.loc.xls
H3K36me3_ChIPSeq_SETD2_KO.bai (S17)
H3K36me3_ChIPSeq_SETD2_KO_INPUT.bai (INPUT_S19)
H3K36me3_ChIPSeq_WT.bai (S16)
H3K36me3_ChIPSeq_WT_INPUT.bai (INPUT_S18)

Genome browser session
(e.g. UCSC)

No longer applicable.

Methodology

Replicates

Kidney cysts from one KM mice and neoplastic masses from one KMS mice were isolated for ChIP-seq analysis, no replications were performed.

Sequencing depth

Total number of reads
H3K36me3_ChIPSeq_SETD2_KO: 61797188
H3K36me3_ChIPSeq_SETD2_KO_INPUT: 78517221
H3K36me3_ChIPSeq_WT_KO: 65177391
H3K36me3_ChIPSeq_WT_KO_INPUT: 65302994
Uniquely mapped reads
H3K36me3_ChIPSeq_SETD2_KO: 42330109
H3K36me3_ChIPSeq_SETD2_KO_INPUT: 58018078
H3K36me3_ChIPSeq_WT_KO: 42435082
H3K36me3_ChIPSeq_WT_KO_INPUT: 48924344
Length of reads: 75
Single or paired-end: single

Antibodies

Primary antibody against H3K36me3 (ab9050) was purchased from abcam. Abcam anti-H3K36me3 antibody (ChIP Grade) is a rabbit polyclonal antibody raised against human and mouse H3K36me3 and is recommended for chromatin immunoprecipitation assay at dilution range 1:200~1:500. (Stewart-Morgan, Kathleen R et al. Nature cell biology, 2023; Barral, Amandine et al. Molecular cell, 2022)

Peak calling parameters

Raw reads were filtered to obtain high-quality clean reads by removing sequencing adapters, short reads (length <50bp) and low quality reads. The clean reads were mapped to the mouse genome (assembly GRCm38). Peak detection was performed with 0.01 set as the p-value cutoff.

Data quality

Cells were cross-linked with 1% formaldehyde for 10 minutes at room temperature and quenched with 125 mmol/L glycine. The fragmented chromatin fragments were precleared and then immunoprecipitated with Protein AþG Magnetic beads coupled with anti-H3K36me3 (ab9050) and immunoglobulin G (Santa Cruz Biotechnology). After reverse cross-linking, chromatin immunoprecipitation (ChIP) and input DNA fragments were end-repaired and A-tailed using the NEBNext End Repair/dA-Tailing Module (E7442, NEB) followed by adaptor ligation with the NEBNext Ultra Ligation Module (E7445, NEB). The DNA libraries were amplified for 15 cycles and sequenced using Illumina NextSeq 500 with single-end 1x75 as the sequencing mode. Raw reads were filtered to obtain high-quality clean reads by removing sequencing adapters, short reads (length < 50 bp), and low-quality reads using Cutadapt (v1.9.1) and Trimmomatic (v0.35). Then FastQC is used to ensure high reads quality. The clean reads were mapped to the mouse genome (assembly GRCm38) using the Bowtie2 (v2.2.6) software. Peak detection was performed using the MACS (v2.1.1) peak finding algorithm with 0.01 set as the P value cutoff.Annotation of peak sites to gene features was performed using the

ChIPseeker R package. A total of 2,723 and 12,199 H3K36me3 peaks were identified in Setd2–KO PTECs and control PTECs at FDR 5% and 5-fold enrichment, respectively.

Software

Cutadapt (v1.9.1)(Anthony M et al., Bioinformatics 2014) and Trimmomatic (v0.35) (Martin M et al., EMBnet.J 2011) were used to remove sequencing adapters, short reads (length <50 bp) and low quality reads. Then FastQC (Andrews S et. al., FastQC 2010) was used to ensure high reads quality. Bowtie2 (v2.2.6) (Langmead B et. al., Nature Methods 2012) software was used to map clean reads to the mouse genome (assembly GRCm38). Peak detection was performed using the MACS (v2.1.1) (Zhang et al., Genome Biol 2008) peak finding algorithm with 0.01 set as the p-value cutoff. Annotation of peak sites to gene features was performed using the ChIPseeker R package (Guangchuang Yu et al., Journal of the Royal Statistical Society Series 1995).
